# Integrated metabolomics and transcriptomics analysis highlight key pathways involved in the somatic embryogenesis of Darjeeling tea

**DOI:** 10.1186/s12864-024-10119-2

**Published:** 2024-02-23

**Authors:** Vivek Kumar Awon, Debabrata Dutta, Saptadipa Banerjee, Soumili Pal, Gaurab Gangopadhyay

**Affiliations:** 1 Department of Biological Sciences, Bose Institute, EN80, Sector V, Salt Lake, Kolkata, 700091 India; 2 School of Agriculture and Food Science, University College Dublin, Dublin, Ireland

**Keywords:** Darjeeling tea, Somatic embryogenesis, Metabolomics, Transcriptomics, Auxin

## Abstract

**Background:**

Darjeeling tea is a globally renowned beverage, which faces numerous obstacles in sexual reproduction, such as self-incompatibility, poor seed germination, and viability, as well as issues with vegetative propagation. Somatic embryogenesis (SE) is a valuable method for rapid clonal propagation of Darjeeling tea. However, the metabolic regulatory mechanisms underlying SE in Darjeeling tea remain largely unknown. To address this, we conducted an integrated metabolomics and transcriptomics analysis of embryogenic callus (EC), globular embryo (GE), and heart-shaped embryo (HE).

**Results:**

The integrated analyses showed that various genes and metabolites involved in the phenylpropanoid pathway, auxin biosynthesis pathway, gibberellin, brassinosteroid and amino acids biosynthesis pathways were differentially enriched in EC, GE, and HE. Our results revealed that despite highly up-regulated auxin biosynthesis genes *YUC1*, *TAR1* and *AAO1* in EC, endogenous indole-3-acetic acid (IAA) was significantly lower in EC than GE and HE. However, bioactive Gibberellin A4 displayed higher accumulation in EC. We also found higher *BABY BOOM* (*BBM*) and *Leafy cotyledon1* (*LEC1*) gene expression in GE along with high accumulation of castasterone, a brassinosteroid. Total flavonoids and phenolics levels were elevated in GE and HE compared to EC, especially the phenolic compound chlorogenic acid was highly accumulated in GE.

**Conclusions:**

Integrated metabolome and transcriptome analysis revealed enriched metabolic pathways, including auxin biosynthesis and signal transduction, brassinosteroid, gibberellin, phenylpropanoid biosynthesis, amino acids metabolism, and transcription factors (TFs) during SE in Darjeeling tea. Notably, EC displayed lower endogenous IAA levels, conducive to maintaining differentiation, while higher IAA concentration in GE and HE was crucial for preserving embryo identity. Additionally, a negative correlation between bioactive gibberellin A4 (GA4) and IAA was observed, impacting callus growth in EC. The high accumulation of chlorogenic acid, a phenolic compound, might contribute to the low success rate in GE and HE formation in Darjeeling tea. TFs such as *BBM1*, *LEC1*, *FUS3*, *LEA*, *WOX3*, and *WOX11* appeared to regulate gene expression, influencing SE in Darjeeling tea.

**Supplementary Information:**

The online version contains supplementary material available at 10.1186/s12864-024-10119-2.

## Background

Darjeeling tea (*Camellia sinensis* (L.) O. Kuntze.) of India is adored worldwide for its unique flavour with aroma. This non-alcoholic beverage also possesses anticancer properties [1]. However, Darjeeling tea genotypes face severe challenges due to their self-incompatibility, precarious seed germination, and abrupt loss of seed viability. Further, vegetative propagation is fraught with issues like cutting degradation, genetic erosion, and disease contamination [2]. These challenges not only limit the production of Darjeeling tea but also warrant the biotechnological exploitation of its crucial economic traits.

SE is an efficient biotechnological tool for clonal propagation and a model system to generate transgenic plants. SE is the formation of bipolar structures from a single or a group of vegetative cells without any vascular link to the original explants. Plant hormones, including auxins, cytokinins (CKs), brassinosteroids, and gibberellins, are crucial in SE [3]. Auxins and CKs are instrumental in determining cell de-differentiation states based on their balance with other hormones in plant tissue culture systems [4, 5]. Specifically, auxins influence the genetic program of somatic cells, guiding them through various stages of SE when combined with other plant growth regulators. As cells align with the SE pathway, the activation of TFs like *BBM*, *SERC*, and *LEC* initiates a series of events, including chromatin remodelling and propelling the induction of SE [4]. Brassinosteroids further influence auxin distribution by modulating genes of auxin exporters, notably *PIN4* and *PIN7*, reflecting their interconnected developmental roles [6].

In tea, SE has been reported from stems and anthers [7], immature leaves [8], and cotyledons [9]. However, the induction of tea somatic embryos rate depends on the explants and genotypes, making tea a recalcitrant plant to manipulate in vitro. The transformation rate from EC to somatic embryos like GE and HE is low [2, 9]. The primary obstacle to improve the SE process in woody plants like tea, cotton, and coffee is the limited understanding of the mechanisms involved in somatic cell reprogramming [10, 11]. Research on SE continues to rely primarily on an empirical approach characterized by low throughput and a trial-and-error method.

However, the integration of diverse omics technologies, such as genomics, transcriptomics, proteomics, and metabolomics, with in vitro processes has the potential to have a profound impact on our understanding of the intricate molecular mechanisms that underlie SE [10, 12, 13]. Mass spectrometry-based global metabolic profiling, which targets a broader spectrum of metabolites, is an emerging platform to explore the metabolic reprogramming of plants during changing developmental stages. Utilizing Liquid Chromatography-Mass Spectrometry (LC-MS/MS), metabolomic profiling has been employed to identify metabolic processes regulating SE in Norway spruce [14] and coffee [15]. Concurrently, the emergence of high throughput transcriptome analysis is highly convenient as it provides a comprehensive gene expression profile of developmental stages to reveal the complex regulatory networks that spatially control cell fate. Recent studies have utilized transcriptome analysis to elucidate the molecular mechanisms of SE in *Arabidopsis* [16], rice [17], cotton [18], *Tilia amurensis* [19], *Hevea brasiliensis* [20] and coffee [21]. In previous study, difference of gene expression pattern in the induced primary calli, as well as re-differentiated roots were investigated during tissue culture of tea plant using transcriptomics only [22].

Due to the limited understanding of the mechanisms involved in somatic embryo development in tea, it is crucial to prioritize the utilization of integrated functional genomics methodologies. These methodologies entail comprehensive profiling of transcripts and metabolites in developing embryos, providing valuable insights to bridge the knowledge gap in this area. As such, our research group sought to remedy this gap by undertaking a comparative metabolomics analysis of EC, GE and HE and further performing transcriptomics analysis to integrate and elucidate the probable regulatory network during SE in Darjeeling tea.

## Results

### Induction of somatic embryogenesis

**Fig. 1:**
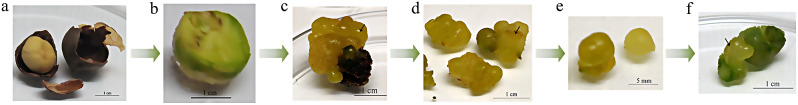
The developmental stages starting from initial explants to heart-shaped embryos in *Camellia sinensis*. (**a**) The seeds split opened from seed coat. (**b**) The cotyledonary explants became yellowish green in induction media after 2 weeks. (**c**) Embryogenic callus with bulges (arrow marked) developed from explants after 4 weeks. (**d**) Embryogenic callus (arrow marked) with globular embryo. (**e**) Separated globular embryos. (**f**) Heart-shaped embryo (arrow marked) developed from globular embryos after 2 weeks

The creamy-white seeds were obtained after removal of brown hard seed coat (Fig. 1a). The cotyledonary explants (Fig. 1b) developed EC in the callus induction medium after 6 weeks of incubation. The EC were light yellow in color, having globular structures on the adaxial surface (Fig. 1c-d). When EC were sub-cultured in the same medium, bulges or globular structures were transformed into GEs after 2–3 weeks. The GEs appeared as yellowish green in color (Fig. 1e) and transformed gradually into greenish HEs within the next 4 weeks (Fig. 1f). However, the transformation rate from globular to heart stage was very low (10–20%).

### Detection of metabolites by LC-MS/MS in developmental stages

The untargeted metabolites of three developmental stages (EC, GE, and HE) were detected through LC-MS/MS. The number of detected metabolites were 647, 658, and 574 in EC, GE, and HE respectively, which fall under broad classes of amino acids, phytohormones, phenylpropanoids, flavonoids and few other metabolites (Table S1). Among 440 differentially accumulated metabolites (DAMs), 168 metabolites were up-regulated and 272 were down-regulated in GE when compared with EC (Table S2). Among 399 DAMs, 227 were up-regulated and 172 were down-regulated in HE when compared with GE (Table S3).

**Fig. 2:**
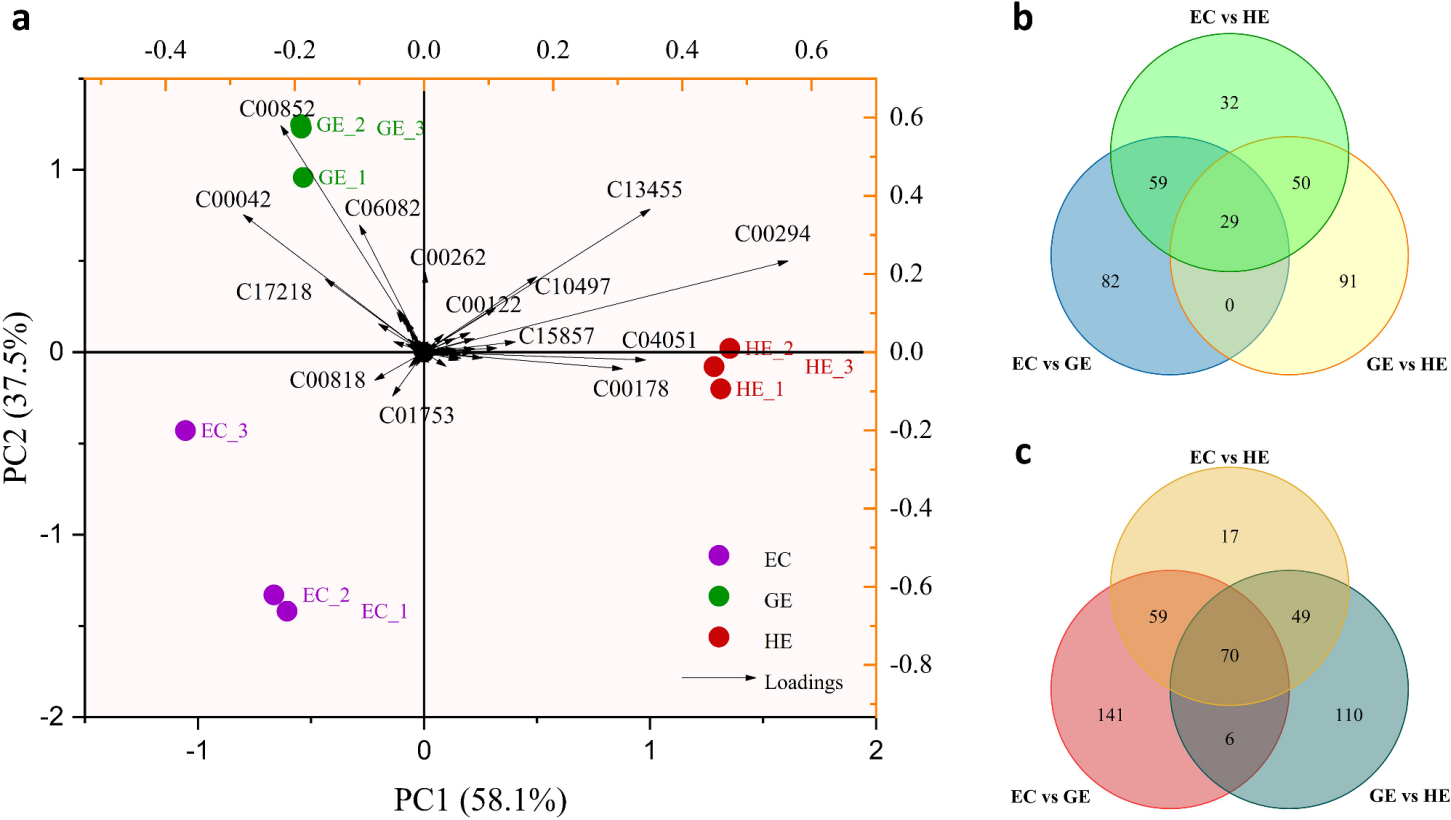
Metabolomics data of embryogenic callus (EC), globular embryo (GE) and heart-shaped embryo (HE). (**a**) Principal component analysis (PCA) (**b**) Venn diagram of up regulated metabolites (**c**) Venn diagram of down regulated metabolites

According to the results of principal component analysis (PCA), first principal component (PC1) accounted for 58.1% of the overall variation in the data set, and PC2 explained 37.5% of the variations present (Fig. 2a). The score-plot obtained from covariance matrix revealed a distinct separation among the three developmental stages. In PCA plot, HE was placed on the positive axis of PC1, which was dominated by the loading of metabolites like abscisic aldehyde (C13455), inosine (C00294), feruloylputrescine (C10497), 5-amino-4-imidazolecarboxyamide (C04051), 9,9’-dicis-zeta-carotene (C15857), 5-methyluracil (C00178) and fumaric acid (C00122), whereas EC and GE were placed on the negative axis of PC1. However, both EC and GE formed two distinct clusters across PC2, where EC falls on the negative axis of PC2 and dominated by the loading of the compounds like D-glucaric acid (C00818), beta-sitosterol (C01753). GE was placed on the positive axis of PC2 and dominated by the loading of compounds like chlorogenic acid (C00852), abscisic acid (C06082), succinic acid (C00042), hypoxanthine (C00262) and 2-(4’-methylthio) butylmalic acid (C17218).

### Analysis of metabolomic differences in EC, GE and HE

**Fig. 3:**
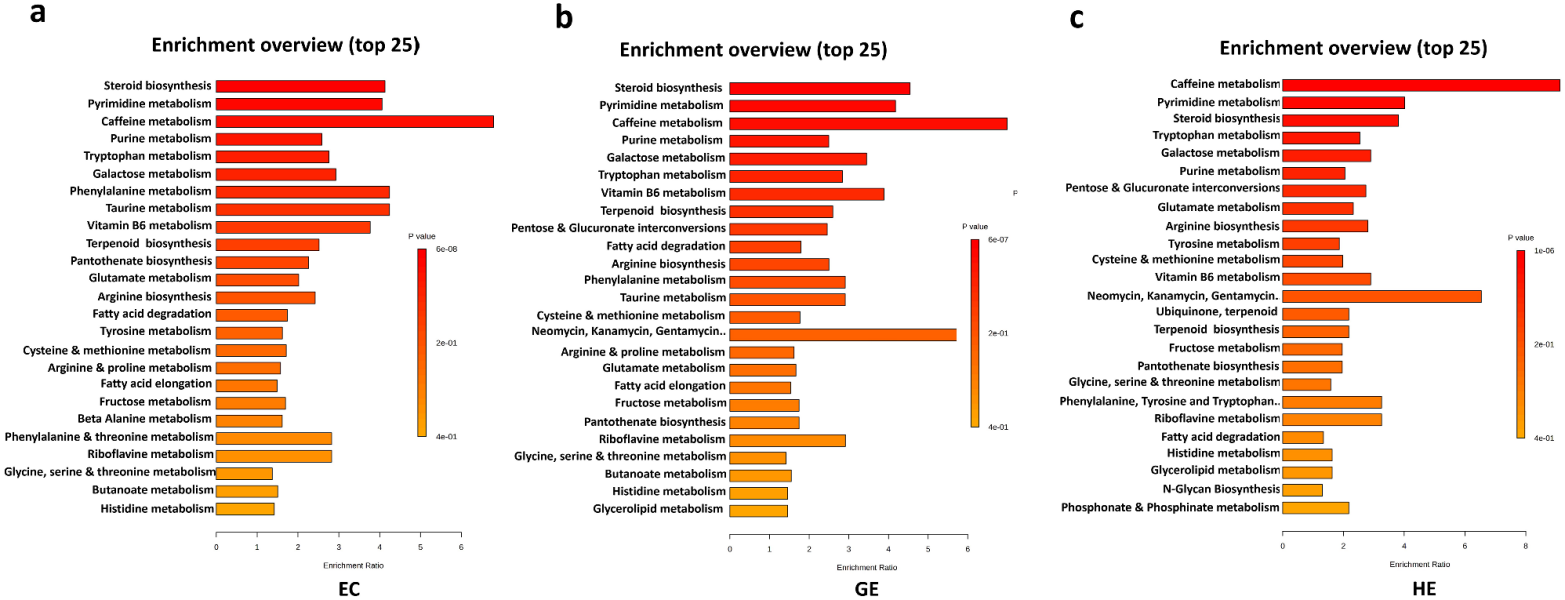
KEGG enrichment analysis of accumulated metabolites in EC, GE and HE

The Venn diagram analysis displayed that 82, 91 and 32 metabolites were specifically up-regulated in EC vs. GE, GE vs. HE and EC vs. HE respectively (Fig. 2b) whereas 141, 110 and 17 metabolites were down-regulated in EC vs. GE, GE vs. HE and EC vs. HE respectively (Fig. 2c). Among the 82 up-regulated metabolites, a few metabolites that showed significant accumulation in GE are gibberellin A12, histidine, sinapoyl-CoA, carbamoyl phosphate, L-aspartic acid when compared with EC. Among 91 up-regulated metabolites, apigenin, L-alanine, glutamic acid, campest-4-en-3-one, luteolin, phenylpyruvic acid, L-tryptophan, gibberellin A53, L-phenylalanine were highly accumulated in HE when compared with GE. Kyoto Encyclopedia of Genes and Genomes (KEGG) enrichment analysis was performed to reveal the enriched metabolic pathways in EC, GE and HE separately. Notably, a substantial convergence in the enriched metabolic pathways exists between EC and GE (Fig. 3a and b). Both EC and GE displayed significant enrichment in steroid metabolism, purine and pyrimidine biosynthesis, phenylalanine metabolism, tryptophan metabolism, and caffeine metabolism pathways. However, a metabolic shift occurs during the HE stages, characterized by a reduction in steroid metabolism and an increase in caffeine metabolism, as well as sharp increase in tyrosine metabolism, cysteine and methionine metabolism (Fig. 3c). The purine and pyrimidine metabolism pathway along with galactose metabolism consistently exhibit enrichment across all developmental stages. Analysis of KEGG enrichment results, utilizing metabolites from three distinct stages, indicates a predominant association with pathways related to plant hormone biosynthesis, primary metabolism of amino acids and sugars, and the biosynthesis of phenylpropanoids from the shikimic acid pathway.

### *De novo* assembly of Darjeeling tea and pathway enrichment analysis from RNA-seq data

RNA-seq analysis of EC, GE and HE yielded a total 139,925,390 clean reads (Table S4). The Q30 of clean reads ranging from 88.83 to 93.61% in six sequenced libraries were *de novo* assembled by Trinity to get transcriptome assembly. On average, 83.28% of clean reads were mapped back to the assembled transcriptome using RSEM software (Table S5). The results indicated the high-quality data satisfying the prerequisites for the subsequent analysis. Unigenes were annotated using seven different databases (NR, NT, PFAM, KOG/COG, Swiss-Prot, GO and KEGG). Out of 174,637 unigenes, 130,006 (74.44%) were annotated in at least one database and 15,615 (8.94%) unigenes were annotated in all seven databases (Table S6). A total number of 11,019 differentially expressed genes (DEGs) were found during comparisons between three developmental stages with a cut off value of|log_2_ (fold change)|>1 for up-regulated genes and|log_2_ (fold change)|< -1 for down-regulated genes having adjusted P-value < 0.05. A total number of 7,039, 1,603 and 2,377 DEGs were observed in EC vs. GE, GE vs. HE and EC vs. HE respectively (Fig. S1). Among 7,039 DEGs in EC vs. GE, 3,785 DEGs were up-regulated and 3,254 DEGs were down-regulated. It was evident that a significantly higher number of genes changed their expression pattern to achieve the GE stage from EC. However, in GE vs. HE, we found only 633 up-regulated DEGs and 970 down-regulated DEGs. Spatial analysis with Venn diagram showed that 701 DEGs were overlapped in different developmental stages but 5,229 DEGs and 517 DEGs were uniquely expressed during EC vs. GE and GE vs. HE respectively (Fig. S2). The top 20 highly expressed DEGs from EC vs. GE, GE vs. HE was shown in Table S7. To further establish the biological functions of DEGs, the Blast2GO analysis was performed using false discovery rate (FDR) adjusted P-value < 0.05 as the cut off. Gene Ontology Database (GO) classification of all the DEGs are mentioned in Table S8. In EC vs. GE, DEGs spanned over 28 GO terms (Figure S3a and S3b) with prominent up-regulated processes including carbohydrate metabolism, transmembrane transport, secondary metabolism and developmental maturation. However, in GE vs. HE transmembrane transport, secondary metabolism and developmental maturation were down-regulated (Figure S3c and S3d).

**Fig. 4:**
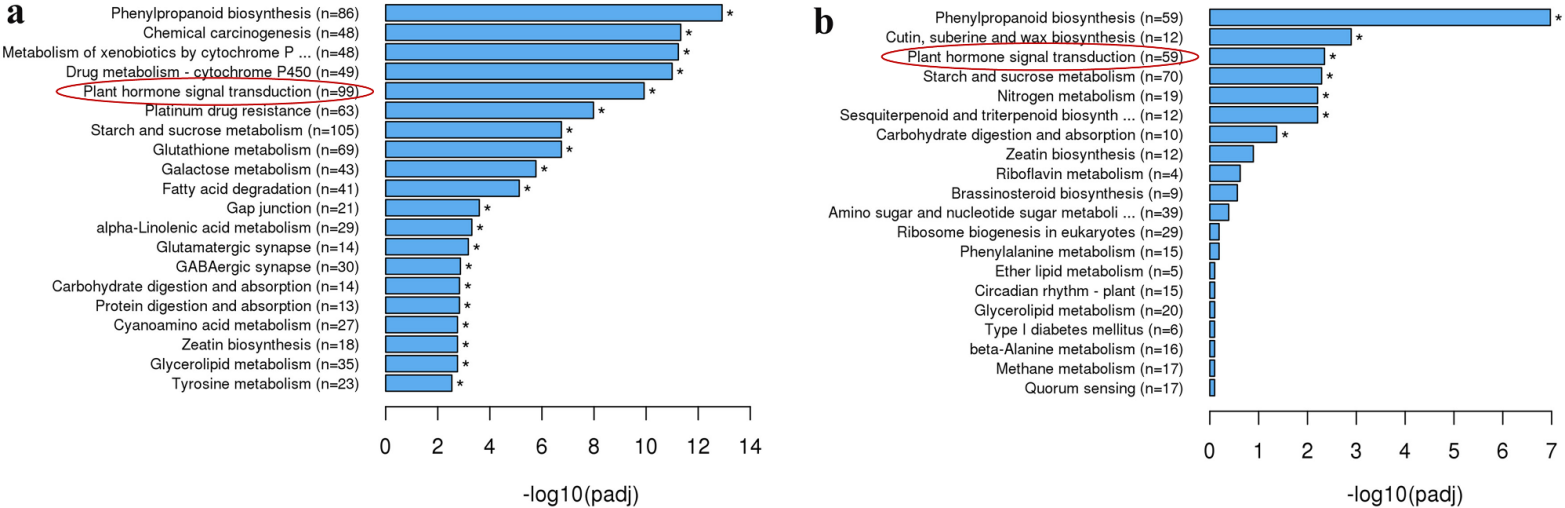
KEGG enrichment analysis of the differentially expressed genes (DEGs) in (**a**) EC vs. GE and (**b**) GE vs. HE. KEGG enrichment was performed with a p-value cut off (FDR) < 0.05. EC: embryogenic callus; GE: globular embryo; HE: heart-shaped embryo

To find out the significant active biological pathways, DEGs were mapped against reference canonical pathways of KEGG using Benjamini and Hochberg method for FDR correction (adjusted P-value <0.05). KEGG pathway analysis showed that 1059 DEGs were enriched in 29 metabolic pathways in EC vs. GE (Fig. 4a). Among 29 metabolic pathways, the significant up-regulated metabolic pathways include phenylpropanoid biosynthesis (86), plant hormone signal transduction (99), starch and sucrose metabolism (105), and zeatin biosynthesis (18). In GE vs. HE, only 241 DEGs were enriched in 7 metabolic pathways (Fig. 4b). The significant up-regulated KEGG pathways include phenylpropanoid biosynthesis (59), and starch and sucrose metabolism (70). The KEGG enrichment analysis of transcriptomics data indicated that multiple physiological processes, notably plant hormone signal transduction, phenylpropanoid biosynthesis and amino acid metabolism had a substantial impact on SE in tea.

### Transcriptional analysis reveals a set of differentially expressed genes involved in auxin biosynthesis

**Fig. 5:**
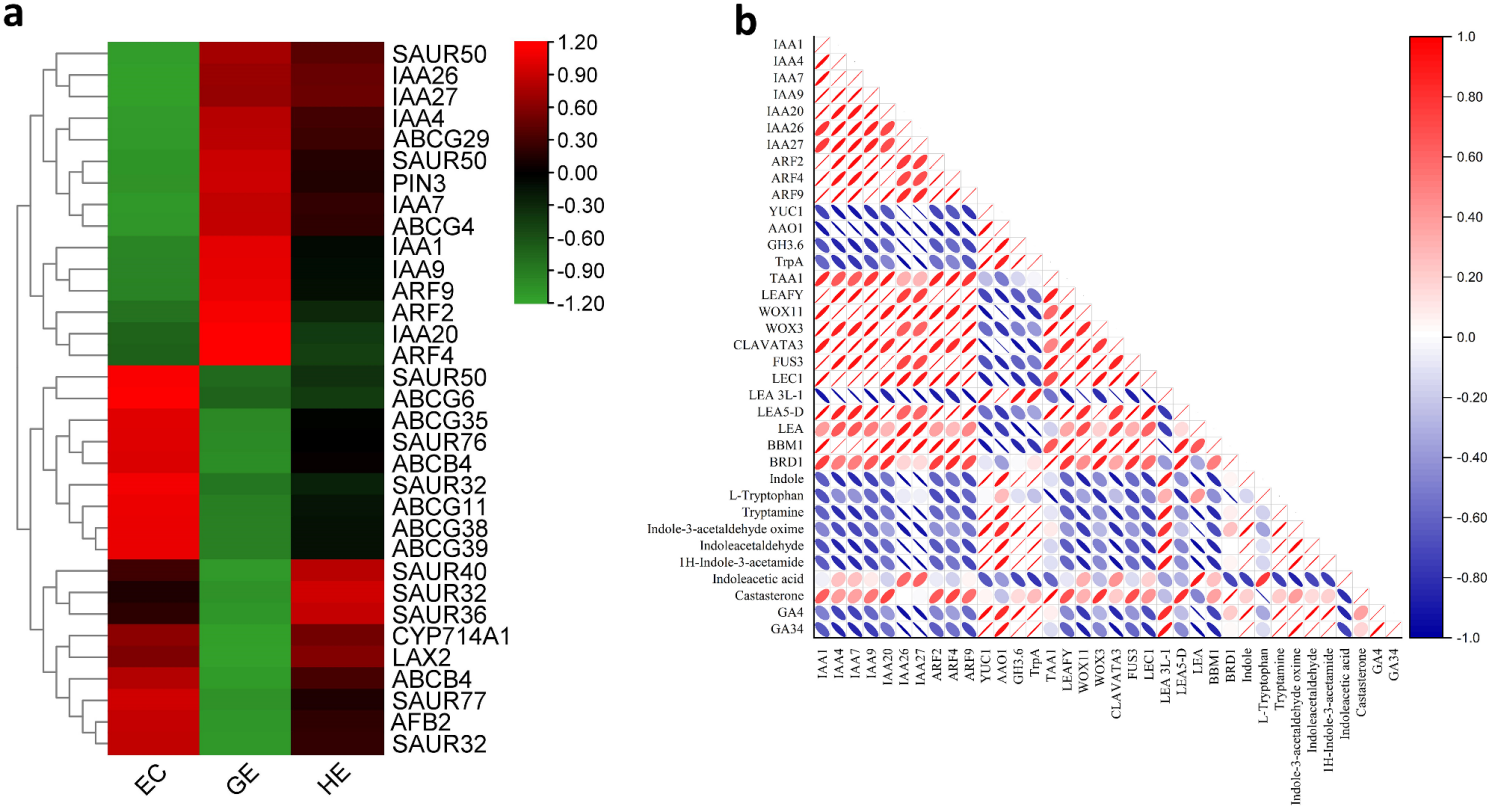
Differentially expressed genes (DEGs) during somatic embryogenesis. (**a**) Heatmap indicates the gene expression level by Log_2_ [FPKM]. (**b**) The Pearson correlation coefficient analysis among differentially expressed *IAA like TFs*, somatic embryogenesis (SE) related TFs, auxin biosynthesis gene, and hormones like indoleacetic acid (IAA), gibberellins and brassinosteroid

Genes involved in auxin biosynthesis like *YUC1*, *AAO1* were highly up-regulated in EC than GE and HE. Auxin transporters *PIN3*, *LAX2*, *ABCB4* transporters exhibited dynamic expression while *ABCG6*, *ABCG11*, *ABCG35*, *ABCG38* and *ABCG39* were mainly expressed in EC. A high number of genes involved in auxin downstream signaling pathways were dynamically expressed in EC, GE and HE. Additionally, auxin early responsive genes like *Gh3.6*, *SAUR32* and *SAUR76* were up-regulated in EC. A heat map of differentially expressed genes involved in auxin transport, auxin signaling, and auxin early responsive genes is provided in Fig. 5a. Multiple sequence alignment of the same genes with different cluster identities (for example, Cluster-17835.140770 and Cluster-17835.140771 for the gene *YUC1*) were performed with Clustal Omega. The result showed maximum alignment rate of 99.85% in *AAO1*, while the minimum was 9.80% in *SAUR32* (Figure S4). Hence, these genes were isoforms having difference in sequences among them.

### Major transcription factors (TFs) and other SE-related genes involved in EC, GE and HE

**Fig. 6:**
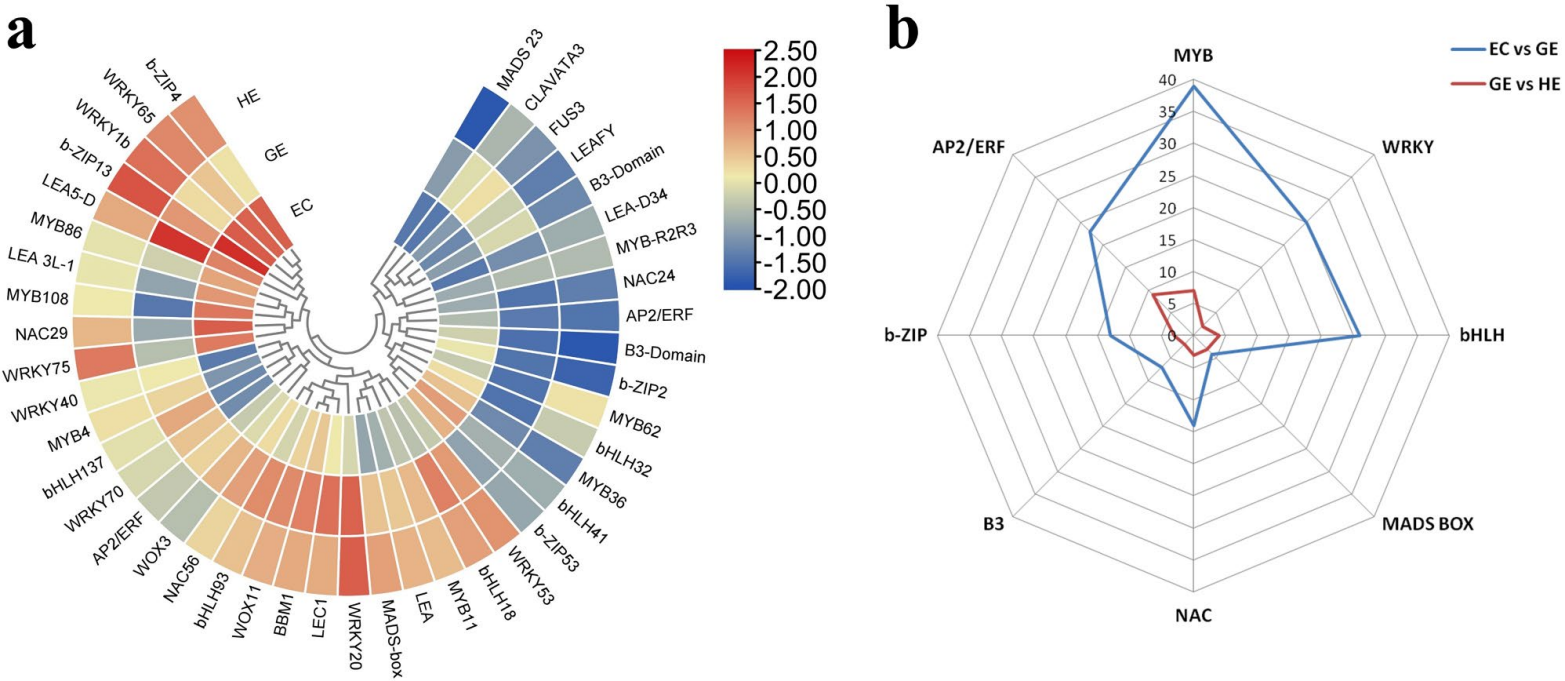
Differentially expressed major transcription factors (TFs), SE-related and histone modification related genes in EC, GE and HE. (**a**) Heatmap of the differentially expressed TFs and SE-related genes. (**b**) Radar chart showing distribution of major TFs in EC vs. GE and GE vs. HE

The TFs play a crucial role in hormone signaling as multifunctional regulators in SE. In auxin signaling pathway, seven *IAA-like* transcription factors, *IAA1-like*, *IAA4-like*, *IAA7-like*, *IAA9-like*, *IAA20-like*, and *IAA27-like* were highly induced in GE. Similarly, three *Auxin Response Factors* (*ARFs*) *ARF2*, *ARF4*, *ARF9* showed higher expression in GE in comparison to EC and HE. The Pearson linear correlation analysis indicated a negative correlation of these TFs with the genes and metabolites associated with auxin biosynthesis (Fig. 5b). This aligns with our hypothesis, as these TFs act as repressors of early responsive auxin genes. We found several other TFs which might not directly involved in hormonal regulation during SE in tea, but were still significantly differentially expressed in EC, GE and HE (Fig. 6a and Table S9). Of the identified TFs, *MYB*, *WRKY*, *bHLH*, *MADS*-box, *NAC*, B3 domain containing transcription factors, *b-ZIP, AP2/ERF* were over-represented. A total of 151 TFs were found in EC vs. GE, while only 33 TFs were found when GE was compared with HE (Fig. 6b). Seven MYB transcripts encoding different MYB TFs displayed complex expression patterns throughout SE. *MYB36*, *MYB86* and *MYB108* were up-regulated in EC while *MYB4* and *MYB11* were induced in GE and HE. *WRKY1b* and *WRKY65* were upregulated in EC, but *WRKY40* and *WRKY70* were down-regulated. Among other crucial SE related TFs, *BBM*, *LEC1*, *WOX3* and *WOX11* were highly expressed in GE and HE in comparison to EC. *LEAFY* and *FUS3* TFs showed a higher expression in GE than EC. When analyzing the correlation between these TFs and IAA, GA4, GA34 catabolite and brassinosteroids, it was observed that *LEA* exhibited a positive correlation with IAA. On the other hand, *WOX3*, *FUS3*, and *LEA5-D* displayed a positive correlation with castasterone, a specific type of brassinosteroid (Fig. 5b). Apart from *LEA3L-1*, all SE-related TFs, such as *LEAFY*, *WOX3*, *WOX11*, *CLAVATA3*, *FUS3*, *LEC1*, *LEA*, and *BBM*, exhibited a negative correlation with GA4 (Fig. 5b).

### Integrated analysis of transcriptome and metabolome for SE

**Fig. 7:**
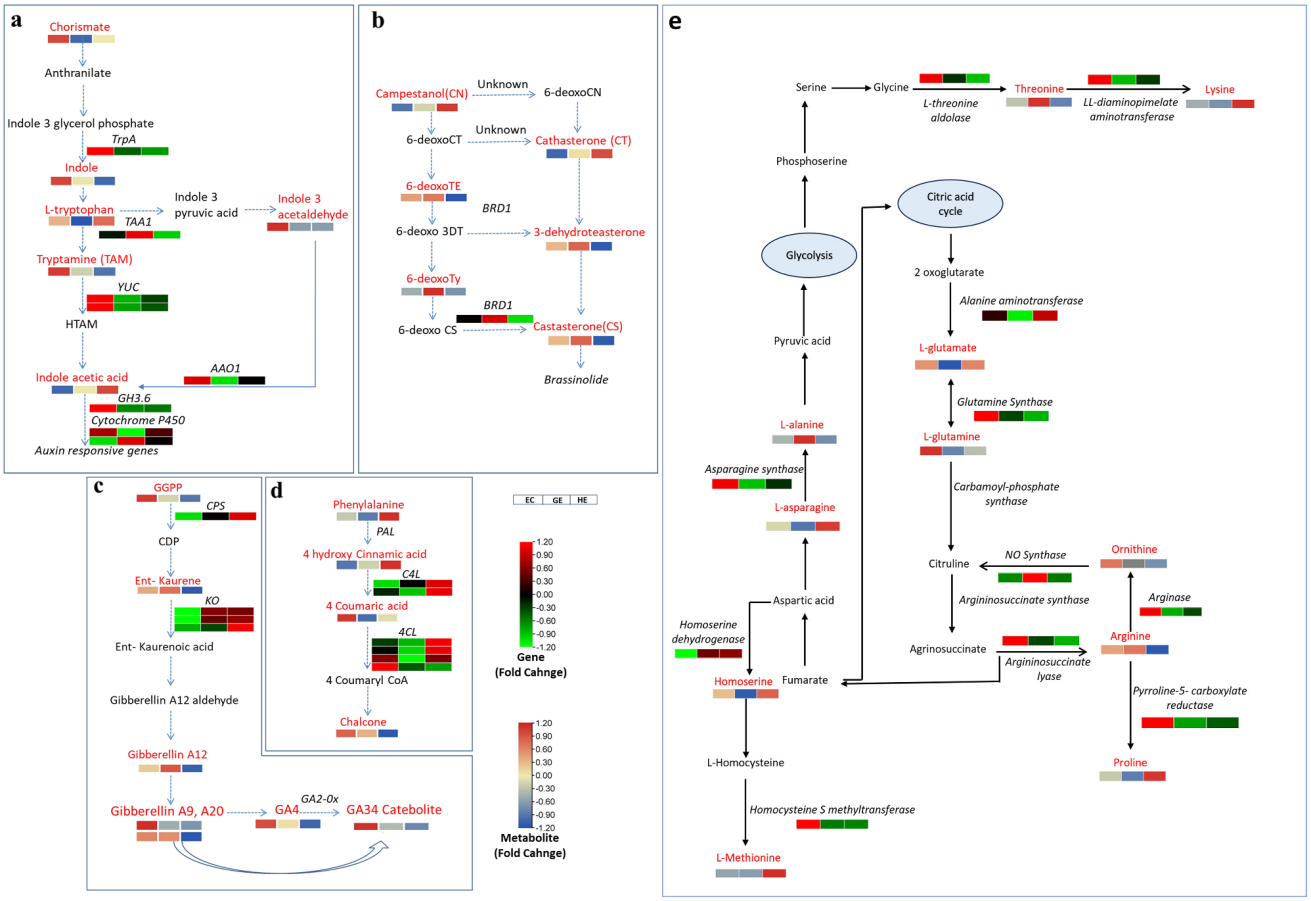
Metabolic pathways involving key differentially accumulated metabolites (DAMs) and differentially expressed genes (DEGs) during somatic embryogenesis (SE) of Darjeeling tea in embryogenic callus (EC), globular embryo (GE) and heart shaped embryo (HE). The pathway maps include (**a**) auxin (**b**) brassinosteroid (**c**) gibberellin (**d**) phenylpropanoid pathway and (e) amino acid metabolism pathways. The small heatmaps illustrate variations in the levels of differentially expressed metabolites (DAMs) and differentially expressed genes (DEGs). The metabolites differentially accumulated are marked in red

Dynamic expression of the genes regulating hormonal signaling pathways determines the somatic embryo development from callus. To investigate the auxin regulation involved in three different stages of SE, the metabolites and DEGs in KEGG pathways related to tryptophan biosynthesis and metabolism, auxin signal transduction were analyzed. The results of the analysis revealed that EC exhibited a substantial enrichment of the tryptophan biosynthesis pathway. The pathway was characterized by significant accumulations of chorismate, indole, and L-tryptophan in EC (Fig. 7a). This observation was further supported by the high expression levels of the *tryptophan synthase* gene in EC, which plays a crucial role in catalyzing the conversion of indole 3-glycerol phosphate to indole. The Pearson linear correlation analysis revealed that tryptamine, indole-3-acetalhdehyde-oxime and indole acetaldehyde were positively correlated with auxin biosynthesis genes like *TrpA*, *YUC1*, *AAO1* (Fig. 5b). The analysis highlighted the significance of *YUC1*, *AAO1*, and *TrpA* genes within the auxin biosynthesis pathway, alongside previously identified *IAA-like* TFs and *ARFs* during SE in tea. Alterations in their expression levels played a crucial role in sustaining either the differentiation stage of EC or preserving the identity of the somatic embryos in tea.

Notably, among other hormones, it is worthwhile to highlight the brassinosteroids biosynthesis and gibberellins pathways. In brassinosteroid biosynthesis pathway, dynamic accumulation of six metabolites, including campesterol, cathasterone, 6-deoxoteasterone, 3-dehydroteasterone, 6-deoxotyphasterol, and castasterone were observed in EC, GE, and HE (Fig. 7b). Notably in GE, 6-deoxocastasterone was found to be highly accumulated, while the gene *brassinosteroid-6-oxidase-1* (*BRD1*), which plays a key role in the conversion of 6-deoxocastasterone to castasterone, was also highly expressed. Accumulation of metabolites associated with GA biosynthesis, such as geranyl geranyl pyrophosphate (GGPP), ent-kaurene, and Gibberellin A12, were present in higher concentrations in EC and gradually declined up to HE (Fig. 7c). Interestingly, the bioactive GA4 and the GA34 catabolite was also significantly enriched in EC. Remarkably, the expression of *DELLA*, a negative regulator within the gibberellin signaling pathway, was notably high in the GE and HE stages but down-regulated in the EC stage. These results imply that SE could be significantly affected by the control of gene expression and the accumulation of metabolites in the gibberellin and brassinosteroid biosynthesis pathways.

Beyond hormonal factors, the phenylpropanoid biosynthesis and amino acid pathways was the top enriched KEGG pathway. We observed that *phenylalanine ammonia lyase* (*PAL*) shows a decrease in expression in EC but an increase in expression in GE. Meanwhile, *4-coumarate co-A ligase* (*4CL*) exhibits significant down-regulation in EC but up-regulation in GE, with even greater up-regulation in HE compared to GE. Among metabolites, phenylalanine, 4-hydroxy cinnamic acid, 4-coumaric acid were differentially accumulated in EC, GE and HE (Fig. 7d). Chlorogenic acid, one of the phenolic compounds was found to be highly accumulated in GE. The main metabolic pathways associated with amino acids exhibited enrichment in terms of DAMs and DEGs (Fig. 7e). Amino acids such as glutamine, lysine, ornithine, proline, asparagine, and homoserine displayed substantial accumulation in EC, followed by a decrease in GE and a subsequent rise in HE. Correspondingly, the genes responsible for the synthesis of these amino acids, such as *delta-1-pyrroline-5-carboxylate synthetase* (involved in proline biosynthesis), *asparagine synthetase*, and *arginase* (involved in the biosynthesis of asparagine and ornithine, respectively), exhibited a similar pattern.

### Validation of RNA-seq data by quantitative RT-PCR

**Fig. 8:**
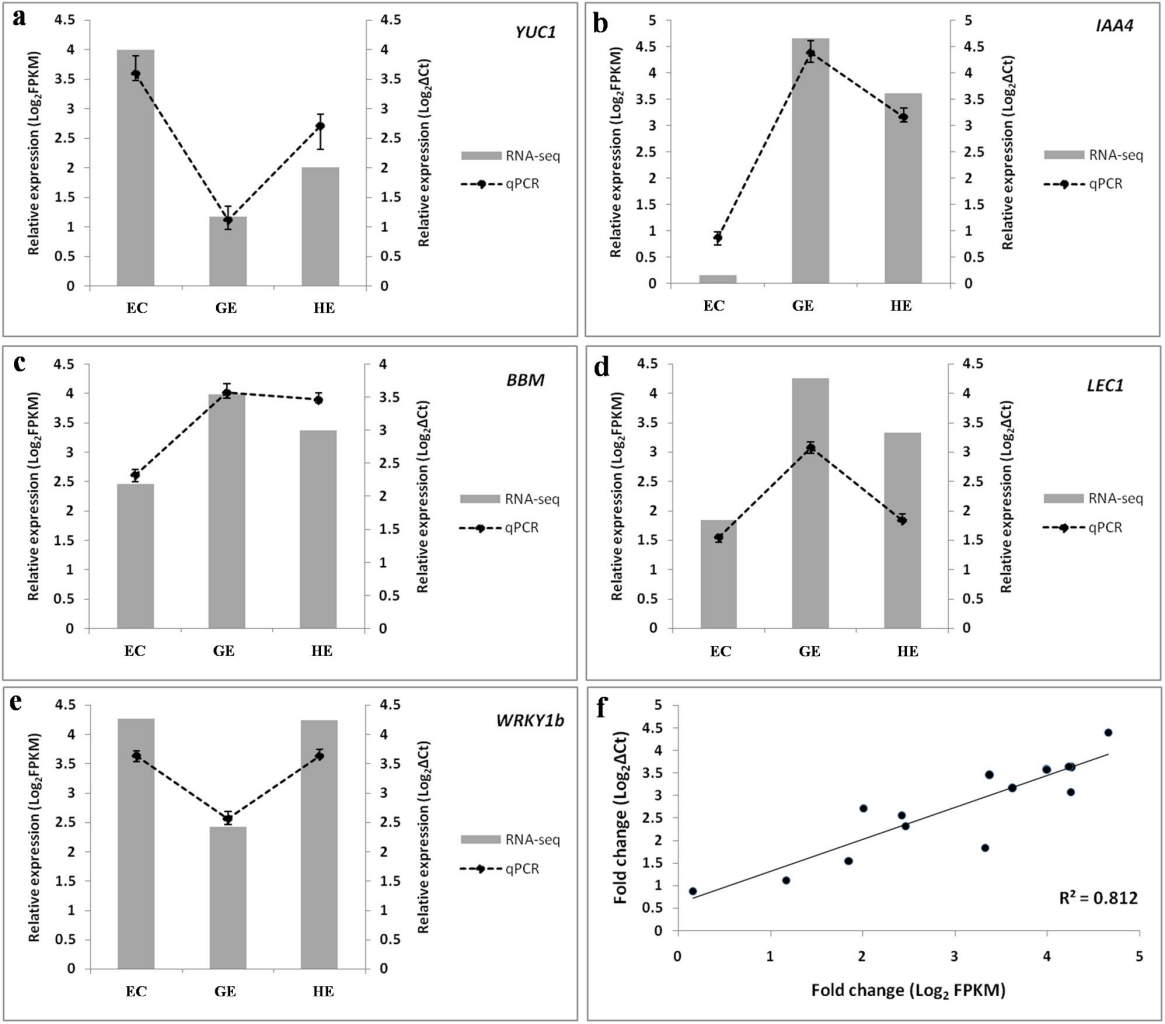
The qRT-PCR verification of the selected DEGs (**a**) *YUC1*, (**b**) *IAA4*, (**c**) *BBM*, (**d**) *LEC1* and (**e**) *WRKY1b* involved in EC, GE and HE. The *Cs26SrRNA* gene from *C. sinensis* was employed as the internal standard for normalization for qPCR. Relative expression levels obtained from qPCR and RNA-seq were shown as polyline and column respectively. The data of polyline and column were derived from 2^^−ΔCt^ and FPKM value respectively. The statistical differences were analyzed by ANOVA (One-way analysis of variance, *P* < 0.05). (**f**) Correlation between RNA-seq data and qRT-PCR results based on the selected genes. EC: embryogenic callus; GE: globular embryo; HE: heart-shaped embryo

To validate RNA-seq results, qRT-PCR was carried out with five randomly chosen genes like *YUC1* (Cluster-17835.140770), *IAA4* (Cluster-17835.10916), *BBM* (Cluster-17835.79421), *LEC1* (Cluster-17835.61481) and *WRKY1b* (Cluster-17835.133594). The *Cs26SrRNA* gene from *C. sinensis* was employed as the internal standard for normalization. The relative expression (2^-ΔCt^) of these selected genes in qRT-PCR was compared with Log_2_ Fragments per Kilobase per Million (FPKM) value of RNA-seq data (Fig. 8a-e). The comparison revealed that the expression trends were highly consistent in both. Further, a good correlation value (r^2^ = 0.81; Fig. 8f) between qRT-PCR and RNA-seq was found to strengthen the reliability of our RNA-seq data.

### Quantification of IAA with HPLC and estimation of total phenolics, flavonoids using UV visible spectroscopy

**Fig. 9:**
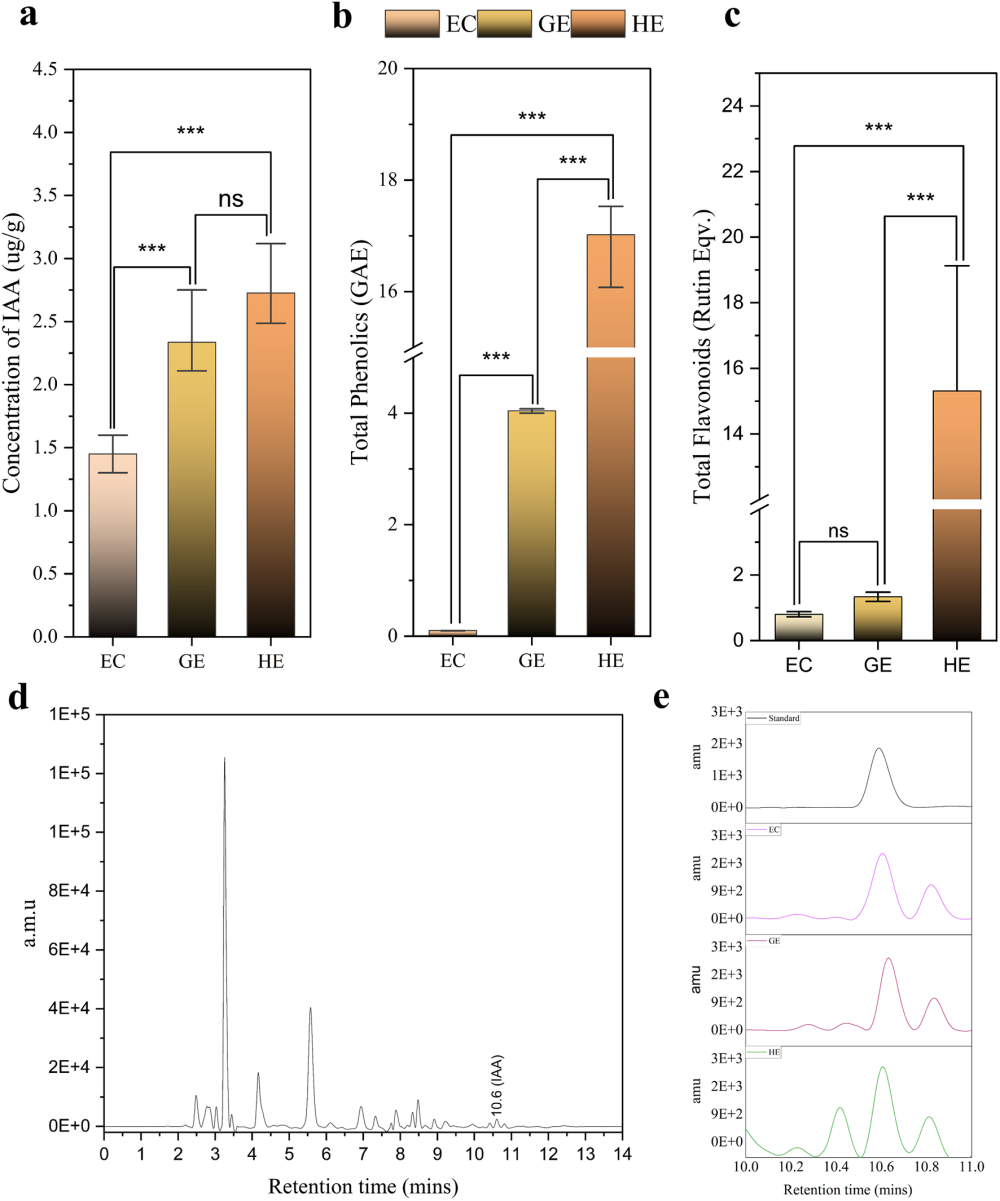
Total content of auxin, phenolics and flavonoids at various stages of SE in Darjeeling tea: (**a**) Quantification of IAA with HPLC, (**b**) estimation of total phenolics and (**c**) flavonoids with UV-vis spectrophotometer. (**d**) HPLC separation of IAA from HE extracts display IAA peak at 10.6 min, (**e**) enlarged chromatogram showing the intensity of IAA in standard, EC, GE and HE. One-way ANOVA analyses were conducted, with *** indicating statistical significance and ‘ns’ indicating non-significance

Analysis using HPLC highlighted a distinct variance in the endogenous IAA levels across EC, GE, and HE, as shown in (Fig. 9a-c). The IAA content showed a steady rise from EC to HE, corroborating our metabolomics findings. EC (1.44 µg/g) had a notably lower IAA concentration compared to GE (2.33 µg/g) and HE (2.72 µg/g). Throughout the three phases of SE, total phenolic accumulation displayed variations. HE (17.01 mg/g) exhibited the peak phenolic levels, whereas EC (0.10 mg/g) exhibited the least (Fig. 9d). Nevertheless, total phenolic content of GE (4.03 mg/g) was markedly greater than EC. Like phenolic content, HE (15.31 mg/g) showed a considerable elevated flavonoid compared to both EC (0.803 mg/g) and GE (1.33 mg/g) (Fig. 9e).

## Discussion

**Fig. 10:**
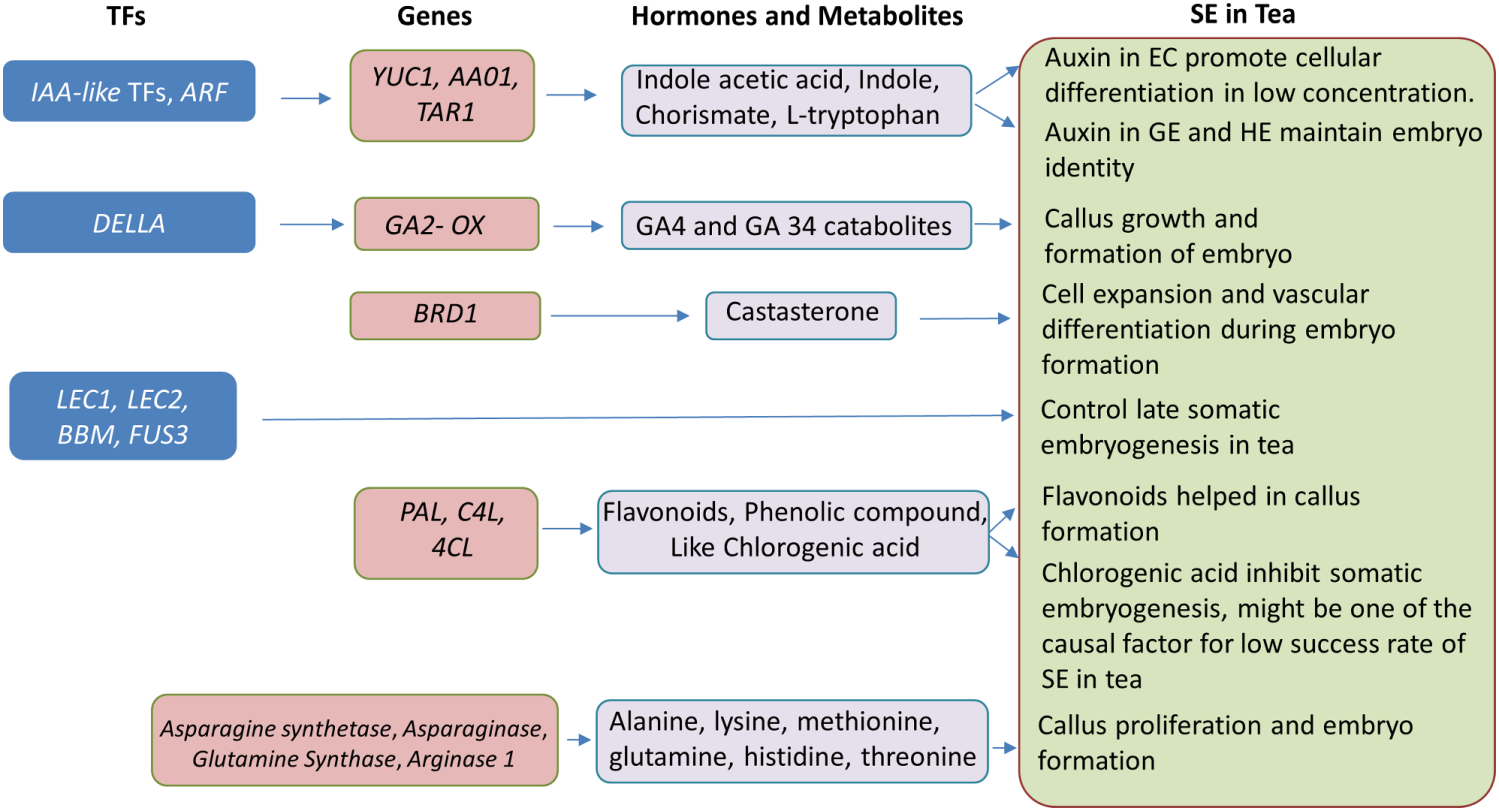
A proposed model depicting somatic embryogenesis mechanism in Darjeeling tea driven by genes and associated metabolites

Darjeeling tea is long overlooked on the front of biotechnology, so exploitation of new approaches appears inevitable to maintain and improve these elite tea varieties. In this context, SE has become increasingly important in tea. However, SE-regulatory network appears to be complicated and it becomes further difficult to understand in woody plants like tea, as it is not extensively studied like *Arabidopsis thaliana*. The goal of the current study was to better understand the probable regulatory network during SE in Darjeeling tea by doing integrated metabolome and transcriptome analysis of EC, GE, and HE. Upon analysis, it was revealed that a multifaceted and intricate network was present, involving diverse hormonal pathways such as auxin, brassinosteroid, gibberellins, and others including phenylpropanoid pathways and amino acid metabolism pathways, indicative of their crucial roles in the development of somatic embryos in Darjeeling tea. A potential model depicting mechanisms driven by transcription and metabolism during SE in Darjeeling tea is illustrated in Fig. 10.

Among the phytohormones, auxin is an essential plant growth regulator, which plays a crucial role in the induction of SE by modulating the genetic program of plant cells [23]. Exogenously applied auxins are essential to regulate somatic embryo development. Despite the applied exogenous auxin stimuli, the up-regulation of auxin biosynthesis genes *YUC1*, *TAR1*, and *AAO1* in EC in our study supports the fact that endogenous auxin biosynthesis is simultaneously crucial. However, the naturally occurring auxin, IAA increased gradually from EC to HE, contradicting the observed pattern of higher expression of auxin biosynthesis genes in EC. It might suggest that in tea, lower concentrations of auxin in EC is necessary to promote cellular differentiation to form successive embryos as prior research suggests that higher concentration of auxin hinder the cellular differentiation [24]. Higher concentration of auxin in GE and HE suggest the crucial role of IAA to maintain the embryo identity in tea. It has been found recently in *Arabidopsis* that an increase in endogenous IAA level is essential to maintain the embryo identity [25]. These observations underscores the critical importance of local auxin biosynthesis and polar auxin transport in the formation of auxin gradients during SE, controlling the appropriate differentiation of cell fates [26–28]. In this study, auxin transporters like *PIN3*, *LAX2*, *ABCB4*, and *ABCG* family transporters exhibited dynamic expressions. These auxin efflux and influx transporters acted cumulatively to maintain the appropriate auxin gradient required for SE development in tea. We observed a significant up-regulation of *Gh3.6* in EC, an early responsive gene to IAA which encode auxin-amido synthetases and facilitate the conjugation of free IAA, thereby contributing to the maintenance of IAA homeostasis [29, 30]. These findings suggest that the increased expression of *Gh3.6* in EC of tea plants may play a role in reducing the endogenous concentration of IAA, possibly aiding in the maintenance of the cellular differentiating stage in EC. Additionally, studies in *Coffea canephora* have revealed the importance of balancing free IAA and conjugated IAA during SE induction [31]. Collectively, it can be concluded that the *Gh3.6* gene might play a critical role to keep the EC in cellular differentiating stage. Among TFs, *IAA-like* TFs and ARF play a pivotal role in auxin signaling. Essentially, *IAA-like* TFs serve as transient regulators, acting as repressors for early auxin response genes when auxin concentrations are low [32]. Observing high expression of these TFs in GE, it can be inferred that *IAA-like* TFs tightly regulate specific auxin-responsive genes in GE. In contrast, the highest concentration of IAA in HE and the downregulation of repressive *IAA-like* TFs compared to GE might suggest a more abundant expression of auxin-responsive genes in HE. The up-regulation of *ARFs* in GE suggests their involvement in mediating embryo axis formation and the differentiation of vascular tissues [33, 34]. All together these TFs actively participate in the auxin signaling pathway, a critical process for SE in tea. Recent studies have revealed the role of gibberellins in formation of embryos from callus during SE in spinach [53]. Our research also implied an increased accumulation of bioactive GA4 in the EC of tea, which might impact callus growth and the formation of embryos from the callus. In brassinosteroid biosynthesis pathway, we found that GE displayed significant up-regulation of *BRD1* and its related metabolite castasterone, a type of brassinosteroid which was positively correlated with *LEAFY*, *WOX3*, *FUS3* and *LEA5-D* (Fig. 5b). Brassinosteroids play a crucial role in cell expansion, root initiation, and vascular differentiation [35]. It is plausible that castasterone might play a pivotal role in cell expansion and vascular differentiation during embryo development in tea. Previous studies have demonstrated that the supplementation of brassinosteroids at suitable concentrations can enhance SE is in plant species like sweetgum [36] and *Coffea arabica* [37].

Among SE related TFs, *BBM* was highly upregulated in GE and HE compared to EC. It has been also found in rice that *BBM* is highly expressed in GEs, and triple knockout of *BBM* genes arrested the embryo formation [38]. Previous studies have also shown that *BBM* can regulate transcription of *LEC1*, *LEC2*, and *FUS3* [24]. Higher levels of *LEC1* expression in GE and HE in our study support earlier results that strongly expressed *BBM* up-regulates *LEC1*, affecting the consequent SE of tea. These results imply that *LEC1* and *BBM* together are major embryonic regulators in tea that might mediate embryogenic programs in cell fate. Recent studies have found that phenylpropanoid pathway plays a crucial role in plant development [39]. Flavonoids and phenolics represent end-products in phenylpropanoid pathway and we found that the levels of total flavonoid and phenolics were elevated in GE and HE. Wang et al. 2018 [40] has reported that knockout of flavonoid synthesis gene *F3H* block callus formation. Conversely, phenolic compounds, including chlorogenic acid, exhibited a significant accumulation in the GE and HE stages. Recent research has demonstrated that these compounds inhibit SE in *C. canephora* by affecting DNA directly or indirectly [41]. These results imply that the elevated accumulation of such phenolic compounds could be a contributing factor to the notably low success rate observed in the formation of GE and HE in tea, as observed in both our studies and previous research [10].

The pattern of amino acid accumulation and the expression of genes associated with amino acid biosynthesis displayed a distinct trend, reaching its peak in EC, decreasing in GE, and then rising again in HE. The heightened amino acid levels in EC may be attributed to the presence of multiple hormones in the nutrient media, as suggested by Feher et al. [42]. According to Sodek et al. [43], the upsurge in amino acid content during the early stages of SE could be linked to the elevated nitrogen concentration in the culture media, serving as the primary material for amino acid biosynthesis. This trend mirrors observations in the SE of *A. sellowiana* [44], where an increase in glutamine accumulation was noted at the callus stage. Similar findings in *Litchi sinensis* indicate the involvement of various amino acids during SE [45]. Overall, the results imply that amino acids may play pivotal roles in tea SE.

Several TFs facilitate SE and its subsequent development. Previous investigations indicated the role of complex transcription regulatory networks sustaining embryogenic competency and embryo development [18, 20]. The *MYB* TFs family is involved in plant development, differentiation and other biological functions [46]. In our study several *MYB* TFs like *MYB36*, and *MYB108* were differentially expressed. The role of *MYB36* in SE is well documented in diverse plant systems [47]. The higher expression of *MYB36* in EC indicates that this TF might have a role during embryo formation in tea. The other TFs found to regulate SE is *bHLH*, among which *bHLH18*, *bHLH32*, *bHLH41*, and *bHLH137* were significantly expressed in different developmental stages of tea. These results suggest the critical role of TFs in SE in tea, as they engage with diverse genes, serving as either transcriptional activators or repressors. Previous studies showed that over-expression of *bHLH109* considerably increased the SE productivity in *Arabidopsis* [48]. Further genome-wide binding study of these SE-related TFs may further interpret their exact role during SE in tea.

SE necessitates a thorough comprehension of the complex interplay between various factors, including the role of auxin in promoting chromatin accessibility and transcriptome alteration [49, 50]. It is achieved through the modulation of chromatin accessibility and remodeling, which subsequently leads to the requisite alterations in the transcriptome necessary for embryogenic competence establishment in somatic cells [51]. Somatic cells decipher the epigenetic process for regulating transcriptional initiation or repression of genes to control developmental plasticity. Histone methyl transferases and histone demethylase are antagonistically acting enzymes that dynamically change the methylation state of histones [52]. The methylation of *H3K4*, *H3K36*, and *H3K79* leads to active transcription, whereas *H3K9*, *H3K27*, and *H4K20* methylations are associated with gene silencing [53]. In our study, genes involved in histone methylation like *SUVH4*, *SUVH5*, and *EZA1* down-regulated significantly in EC compared with GE and HE. The *SUVH4* and *SUVH5* methylate ‘Lys-9’ of histone H3, whereas *EZA1*, a polycomb group (PcG) protein methylates ‘Lys-27’ of histone H3 to employ the transcriptional repression [54, 55]. The down-regulation of these histone-repressive marks in EC suggests the de-condensation of chromatin, which directly influences transcriptional activation. Previous studies have also established that the methylation state of histone-repressive signatures H3K9me2 and H3K27me3 decreased in *Coffea canephora* depending on embryo development [41]. A histone-lysine N-methyltransferase, *ATXR7*, was highly induced in EC, which methylated ‘Lys-4’ and ‘Lys-36’of histone H3 to initiate the transcription. Probably these histone modifications are cumulatively necessary to maintain the high transcriptional rate for cellular reprogramming in EC. In contrast, histone demethylation genes like *JMJ25* were highly expressed in GE, which demethylates ‘Lys-9’ of histone H3 to control several developmental processes by protecting genes from silencing [56]. These histone modification genes might have significant functions during the embryogenesis of tea. S-adenosylmethionine (SAM), a critical cofactor required for a diverse range of methylation reactions, has been thoroughly investigated as a primary substrate or cofactor for enzymes involved in chromatin modification [57, 58]. Our research reveals a notable elevation in the concentration of SAM in EC compared to GE and HE. This observation underscores the crucial role of various gene transcription and silencing events in the EC stage as they determine the fate of proliferating cells and their subsequent transformation into a proper embryo. However, in vitro conditions (supplemented growth regulators in media, environment, and stress response) can often change histone modification [20]. Hence, it is unclear whether the changes in the transcript level of histone modification-related genes are due to developmental changes or in vitro conditions.

## Conclusion

The present study is the first attempt to explore the underlying metabolite regulatory network during SE through precise embryo-stage specific metabolomics and transcriptomics approaches in Darjeeling tea. Through association analysis of enriched metabolic pathways, we found that SE in Darjeeling tea involves essential components, mainly auxin biosynthesis-signal transduction, brassinosteroid, gibberellin, phenylpropanoid biosynthesis, amnio acids and TFs. Our results revealed that EC exhibits lower endogenous levels of IAA, while HE shows higher concentrations. This implies that the lower IAA concentration in EC is conducive to maintain its differentiation stage, whereas higher IAA levels are crucial for preserving the embryo’s identity. Contradicting to IAA, it was found that the bioactive GA4 was negatively correlated with auxin. Higher accumulation of GA4 in EC might impact the callus growth and the formation of embryos from the callus in tea. In contrast, high accumulation of phenolic compound chlorogenic acid might contribute to the notably low success rate observed in the formation of GE and HE in tea. TFs like *BBM1*, *LEC1*, *FUS3*, *LEA*, *WOX3* and *WOX11* may regulate the expression of genes and metabolites accumulation, and all these processes appeared to contribute to SE in tea. In addition, the comprehensive dataset generated through this research will significantly contribute to the transcriptomics and metabolomics resources available for Darjeeling tea, thereby providing a foundation for further advancements in tea research.

## Materials and methods

### Plant material

Green capsules of an elite Darjeeling tea (*Camellia sinensis* (L.) O. Kuntze. var. T-78) were collected from the Makaibari Tea Estate (26°50’37.67"N, 88°16’0.85"E), Darjeeling, India during June-September 2019. This elite tea variety is maintained by Darjeeling Tea Research and Development Center (DTRDC), Kurseong and was collected in accordance with institutional, national, and international regulations and recommendations. Seed coats were surface disinfected with 0.5% HgCl_2_ for 15 min followed by five times rinsing with sterile distilled water. It was further sterilized with 75% (v/v) ethanol followed by flaming. After removal of the hard seed coats, embryonal axis was carefully excised from cotyledons. The cream-colored cotyledons were sliced and used as explants. The explants were cultured in MS basal medium supplemented with 10 mg L^− 1^ 6-benzylaminopurine (BAP), 0.5 mg L^− 1^ indole-3-butyric acid (IBA), 50 mg L^− 1^ adenine hemi-sulphate, 20 g L^− 1^ sucrose and 7 g L^− 1^ agar. The pH of the medium was adjusted to 5.6 and sterilized at 120 °C, 15 psi for 15 min. The explants were kept at 24 ± 1 °C under 16/8 light/dark period. The embryogenic callus developed from explants after 6 weeks of incubation with visible appearance of bulges. After 2–3 weeks of sub-culture in the same medium, the bulges started to develop into GEs. Individual GEs were selected and cultured on fresh medium of the same composition. Within next 4 weeks, the GEs gradually converted into HEs. The EC, GE and HE were instantly frozen in liquid nitrogen and kept at -80 °C until metabolites and RNA extraction.

### Sample extraction and metabolite profiling

The powdered samples of each developmental stage (100 mg) were separately pulverized in liquid nitrogen and extracted with 1 ml of 70% (v/v) methanol. The macerates were mixed for 1 min in a cyclo-mixer, sonicated at 4 °C for 20 min, followed by centrifugation at 12,000 rpm at 4 °C for 10 min. Finally, the supernatants were filtered through 0.22 μm membrane and decanted into glass vials, ready to be used for metabolomics analysis. Metabolite profiling was performed using a Waters ACQUITY UPLC M-Class system (Waters Corporation, Miliford MA, US) system equipped with an Electrospray Ionization Source (ESI, model Xevo G2-XS Q-Tof MS/MS). Chromatographic separation was performed through a UPLC-BEH C18 column (Pore size 130 Å, particle size 1.7 μm, inner diameter 2.1 mm x length 100 mm). The sample injection volume was 10 µl and the temperature of the auto-sampler was 4 °C. The Mass Spectrometry (MS) data was collected in an electrospray ionization-positive (ESI+) mode, described by the following parameters: cone flow temperature 50 L/h; desolvation gas flow 400 L/h; spray voltage of 3.0 kV in ESI+ (3 kV; source temperature 100 °C; desolvation temperature and S-Lens RF level of 30% in ESI+. The mass spectra scan range was set as m/z 50-2000. Quality control (QC) samples were used to assess the reproducibility and reliability of the LC-MS system. QC samples were prepared by pooling an aliquot of all the samples (10 µl) from each sample for analysis.

The open source MZmine3 software was used to process and analyze the generated LC-MS/MS spectra [59]. The mass spectra obtained at each time were used to provide a preliminary identification of the metabolites. The final masses in the mass feature list were identified using the Metaboanalyst 5.0 [60] server after duplicate signals of K^+^, Na^+^, and NH_4_^+^ ions, isotope signals, and fragment ion signals were removed. Mass intensities also derived from MZmine3 were considered for the quantitative assessment of metabolites.

### Statistical analysis of metabolomic data

After checking data integrity, spectral binning, data normalization and annotation (compound ID), markedly changed metabolites were arranged based on their Log_2_ fold changes and p values (*p* < 0.05). Metabolites exhibiting a Log_2_ fold change > 1 were regarded as significantly upregulated, whereas metabolites displaying a Log_2_ fold change <-1 were deemed notably downregulated. Multiple testing was controlled using the FDR. The scaled datasets resulting from the study were further uploaded to the MetaboAnalyst 5.0 server for multivariate statistical analysis. PCA was conducted to verify the consistency between the three biological replicates at each developmental stage. Pathway analysis was carried out on the significant metabolites using *Arabidopsis thaliana* pathway libraries within MetaboAnalyst 5.0 server (https://www.metaboanalyst.ca).

### RNA extraction, library preparation and sequencing

Total RNA was extracted using Spectrum™ Plant Total RNA Kit (Sigma-Aldrich, Germany) from EC, GE and HE. The experimental design for consideration of biological replica was followed, as described by Assefa et al. [61] and Dasgupta et al. [62]. Briefly for RNA-seq, RNA from identical samples of each stage (EC/GE/HE) was pooled separately and considered as a single biological replicate. Two such biological replicates were used. Initially, the purity and concentration of each RNA sample were analysed by NanoDrop (Thermo Scientific, USA). Later, the RNA integrity (RIN) was checked using RNA Nano6000 Assay Kit of Agilent Bioanalyzer 2100 system (Agilent Technology, CA, USA). Following the quality control procedures, oligo (dT) beads were used to enrich mRNA from total RNA. The mRNA was fragmented at random using divalent cations in NEBNext® First Strand Synthesis Reaction Buffer (5X). Transcriptome libraries were created using the NEBNext®Ultra™ RNA Library Prep Kit for Illumina® (NEB, USA) according to the manufacturer’s instructions. The Agilent Bioanalyzer 2100 system was used to assess the quality of libraries. PE Cluster Kit cBot-HS was used to cluster the index-coded samples using the cBot Cluster Generation System (Illumina). The preparation and sequencing of cDNA libraries were performed at Eurofins Genomics, India using the Illumina Novaseq6000 platform generating 139.96 million paired end reads with an average size of 150 bp.

### *De novo* transcriptome analysis

Raw reads that passed Illumina’s quality control, were processed through in-house perl scripts in FASTQ format. To obtain clean data, the low-quality reads containing adapter and poly-N were removed from the raw data. *De novo* transcriptome assembly was accomplished with clean reads using Trinity [63] with ‘min-kmer-cov’ set to 1 and all other parameters set to default. To achieve comprehensive gene function, all unigenes were annotated against following seven databases: NR, NT, PFAM database (protein family), KOG, Swiss-Prot database, KEGG, and GO. The mapping result of Bowtie, analyzed with RNA-Seq by Expectation-Maximization (RSEM) to get the read count for each gene of each sample, was converted into FPKM value. Before the differential gene expression analysis, for each sequenced library, the read counts were adjusted by edgeR program package through one scaling normalized factor. The intensity of the gene expression was measured in FPKM. Differential expression analysis was performed using the DEGseq R package [64]. P-value was adjusted using q value < 0.005. The|log_2_ (fold change)| >1 was set as the threshold for significantly up-regulated and|log_2_ (fold change)| < -1 were deemed as notably down-regulated genes and TFs. The heat map was generated with the TBtools using log_2_ [FPKM] [65].

### Quantitative real-time PCR analysis

To validate the gene expression results obtained from the RNA-seq, qRT-PCR analysis was performed with RNA samples where each sample had three biological replicates. First cDNA strand was synthesized using the QuantiTect® Reverse Transcription kit following the manufacturer’s (QIAGEN, Germany) protocol. The qRT-PCR analysis was performed using Maxima SYBR Green/ROX qPCR mix (Thermo Scientific, USA) with gene-specific primers in an Applied Biosystems® 7500 fast real-time PCR machine. The *Cs26SrRNA* (*Camellia sinensis* 26S rRNA) gene was used as an internal reference gene for normalization [66]. Primer–blast was used to design primers with the expected amplicon size 100–250 bp using default parameters. The primer pairs have been provided in Table S10. PCR cycle was carried out as follows: 40 cycles of 95 °C for 15 s, 60 °C for 30 s and 72 °C for 30 s. The formula of 2^−ΔCt^ was used to calculate the relative expression levels of selected genes [67].

### Quantification of endogenous IAA contents

To quantify the endogenous IAA, we followed the method described by Shah et al. [68] with minor modification in mobile phase gradient. From EC, GE and HE, 250 mg of tissue was crushed in liquid nitrogen and extracted in 1 ml of HPLC grade ethanol respectively. After centrifugation (10,000 g for 20 min at 4°C), 700 µl was collected and filtered through 0.22 μm filter membrane and transferred to glass vials. HPLC (Shimadzu, HPLC) analysis was performed having Shim-pack GWS (Shimadzu) C18 column (4.6 × 250 mm, 5 μm), equipped with SPD-20A Detector and LC-20AD Binary Pump. Gradient concentration of mobile phase was 80–40% acetonitrile for 0.1 min; 40–80% acetonitrile for 7 min; 80% acetonitrile for 15 min. 0.1% acetic acid in water flow rate was 1 ml/min and 10 µl of sample was injected. Absorbance of IAA was recorded at 280 nm. Identification of IAA peak was identified by comparing the peaks using co-chromatography with authenticate IAA standard maintaining same conditions. IAA peaks were estimated by calibrating against standard concentration of IAA between 0.3 µg/ml to 5 µg/ml with 6 different concentration gradients (r^2^ = 0.998; equation y = 39555x + 5.4187). The concentration of the IAA is represented as µg/g as calculated from the fresh weight of EC, GE and HE.

### Estimation of total phenolics and flavonoids using UV vis spectrophotometer

The total phenolic content from EC, GE and HE were estimated following the protocol of Ainsworth et al. [69]. Briefly, 20 µl of the methanolic extract and 40 µl of 10% (v/v) folin-ciocalteu solution was mixed thoroughly and kept in the dark for 5 min. Methanol (95% v/v) was used as the blank. To each tube, 160 µl of 700mM Na_2_CO_3_ was added and incubated in the dark for 2 h. The absorbance of the reaction mixture was measured at 765 nm and quantified from the standard curve prepared using gallic acid equivalents (GAE). The protocol by Csepregi et al. [70] was employed to assess the total flavonoid content. Initially, 20 µl of methanolic extract was mixed with 60 µl of millipore water. This was then supplemented with 6 µl of 5% sodium nitrite, stirred well, and allowed to sit at room temperature for 5 min. Subsequently, 6 µl of 10% aluminium chloride was incorporated into the existing mixture and left undisturbed for another 5 min at room temperature. As a final step, 40 µl of 1mM NaOH was added to the mixture, along with 68 µl of distilled water to achieve a total volume of 200 µl. The mixture was then set aside for 15 min at room temperature. The absorbance was taken at 510 nm, and the total flavonoid content was determined from the standard curve prepared using rutin hydrate equivalents.

### Electronic supplementary material

Below is the link to the electronic supplementary material.

**Supplementary Material 1: Fig S1.** The number of up and downregulated differentially expressed genes (DEGs) in EC vs. GE, GE vs. HE, EC vs. HE, and sum of them. EC: embryogenic callus; GE: globular embryo; HE: heart shaped embryo

**Supplementary Material 2: Fig S2.** The Venn diagram of differentially expressed genes in EC vs. GE, GE vs. HE and EC vs. HE developmental stages of Camellia sinensis. The number of Differentially expressed genes (DEGs) present in multiple processes is represented by the overlapping regions. The expressed genes in all the three comparative groups displayed in the center portion. EC: embryogenic callus; GE: globular embryo; HE: heart shaped embryo

**Supplementary Material 3: Fig. S3.** Gene ontology (GO) analysis of differentially expressed genes (DEGs) in biological processes (BP), cellular component (cc) and molecular function (MF) categories. (a) Upregulated GO enriched DEGs in EC vs GE. (b) Downregulated GO enriched DEGs in EC vs GE. (c) Upregulated GO enriched DEGs in GE vs. HE. (d) Downregulated GO enriched DEGs in GE vs. HE. Go enrichment was performed with a p-value cut off (FDR) <0.05. EC: embryogenic callus; GE: globular embryo; HE: heart shaped embryo

**Supplementary Material 4: Fig. S4.** Multi-sequence alignment of amino acid sequences of similar genes

**Supplementary Material 5: Table S1.** Total detected metabolites in EC, GE and HE

**Supplementary Material 6: Table S2.** Top 272 Down regulated and 168 up regulated metabolites in GE vs EC

**Supplementary Material 7: Table S3.** Top 227 Up regulated and 172 down regulated metabolites in HE vs GE

**Supplementary Material 8: Table S4.** Quality control statistics of Darjeeling tea transcriptome

**Supplementary Material 9: Tables S5-S6.** Summary for the clean reads mapped to the Trinity-assembled transcriptome and summary statistics of transcriptome annotation

**Supplementary Material 10: Table S7.** Top 20 highly expressed DEGs in EC vs GE and GE vs HE

**Supplementary Material 11: Table S8.** EC vs GE GO enrichment up-regulated; EC vs GE GO enrichment down-regulated; GE vs HE GO enrichment up-regulated and GE vs HE GO enrichment down-regulated

**Supplementary Material 12: Table S9.** FPKM and annotation of transcription factors, SE-related genes and histone modification related genes during somatic embryogenesis of Darjeeling tea

**Supplementary Material 13: Table S10.** The particulars of the gene-specific primers used for qRT-PCR validation. Standardized annealing temperature (Ta) was 60 °C for all reactions

## Data Availability

The original RNA-seq data sets generated and analyzed for this current study are available in the NCBI Sequence Read Archive repository under the accession number of PRJNA796008 (https://dataview.ncbi.nlm.nih.gov/object/PRJNA796008?reviewer=f7cqfji7v22068vn49t1hu4bt0).

## Abbreviations

SE: Somatic embryogenesis
DEGs: Differentially expressed genes
DAMs: Differentially accumulated metabolites
EC: Embryogenic callus
GE: Globular embryos
HE: Heart-shaped embryos
*BBM*: BABY BOOM
*LEC1*: Leafy cotyledon
NR: NCBI non-redundant protein sequences
NT: NCBI nucleotide sequences
PFAM: Protein family
KOG: Eukaryotic orthologous groups
COG: Clusters of orthologous groups of proteins database
GO: Gene ontology
KEGG: Kyoto encyclopedia of genes and genomes
FDR: False discovery rate
FPKM: Fragments per kilo base of transcript sequence per million base pairs sequenced
*ARF*: Auxin response factor
TFs: Transcription factors
*AP2/ERF*: Apetala2/Ethylene response factor
*LEA*: Late embryogenesis abundant protein
MS: Murashige and Skoog
BAP: 6-benzylaminopurine
IBA: Indole-3-butyric acid
FC: Fold change
cDNA: Complementary DNA
*HATs*: Histone acetyltransferases
