# Supplementary figures and images for: Integrated metabolomics and transcriptomics analysis highlight key pathways involved in the somatic embryogenesis of Darjeeling tea
Source: BMC Genomics. 2024 Feb 23;25:207. doi: 10.1186/s12864-024-10119-2 (PMC10893738; doi:10.1186/s12864-024-10119-2)

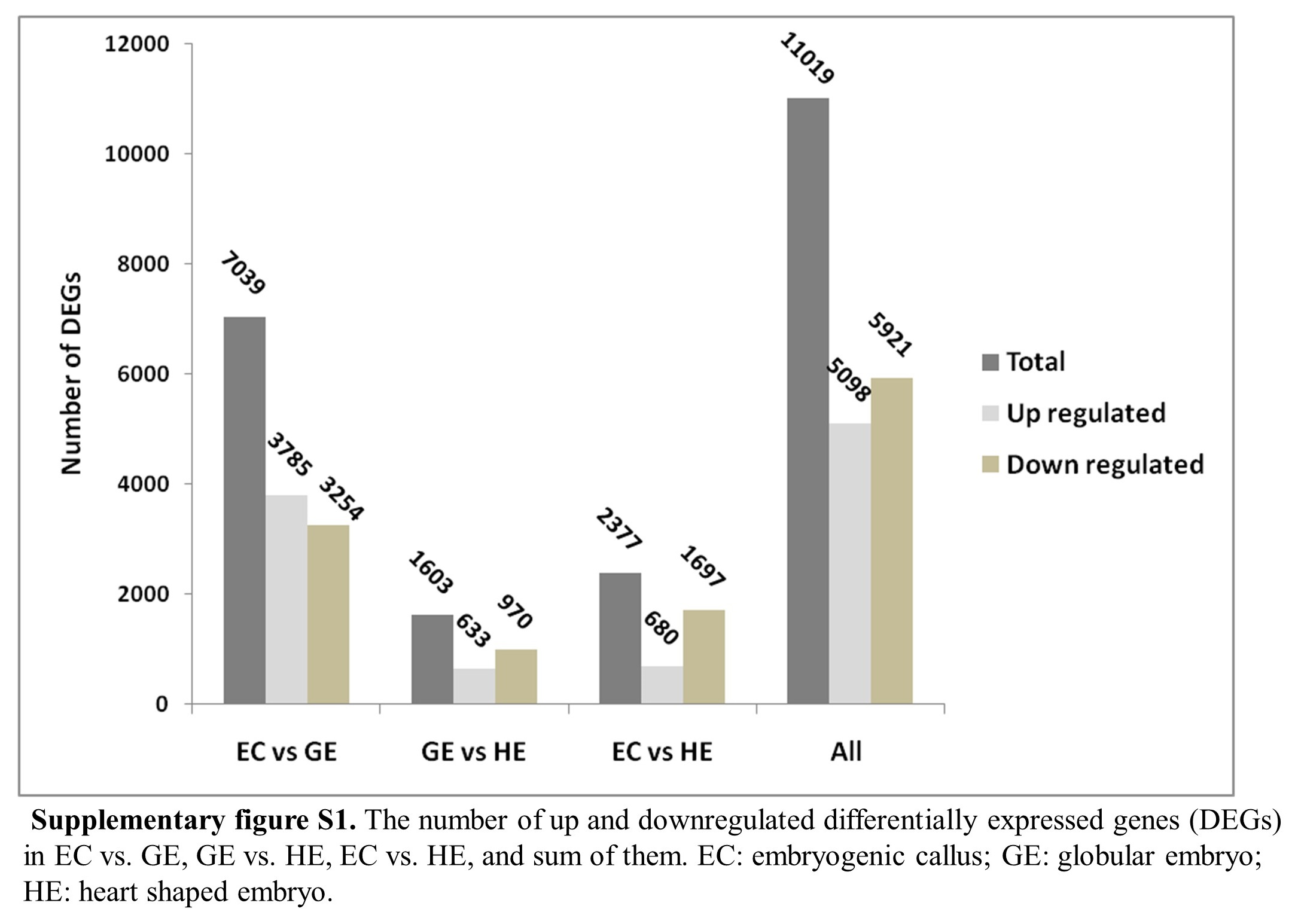

Supplement: Supplementary file 1 — Supplementary Material 1: Fig S1. The number of up and downregulated differentially expressed genes (DEGs) in EC vs. GE, GE vs. HE, EC vs. HE, and sum of them. EC: embryogenic callus; GE: globular embryo; HE: heart shaped embryo [file 12864_2024_10119_MOESM1_ESM.jpg]

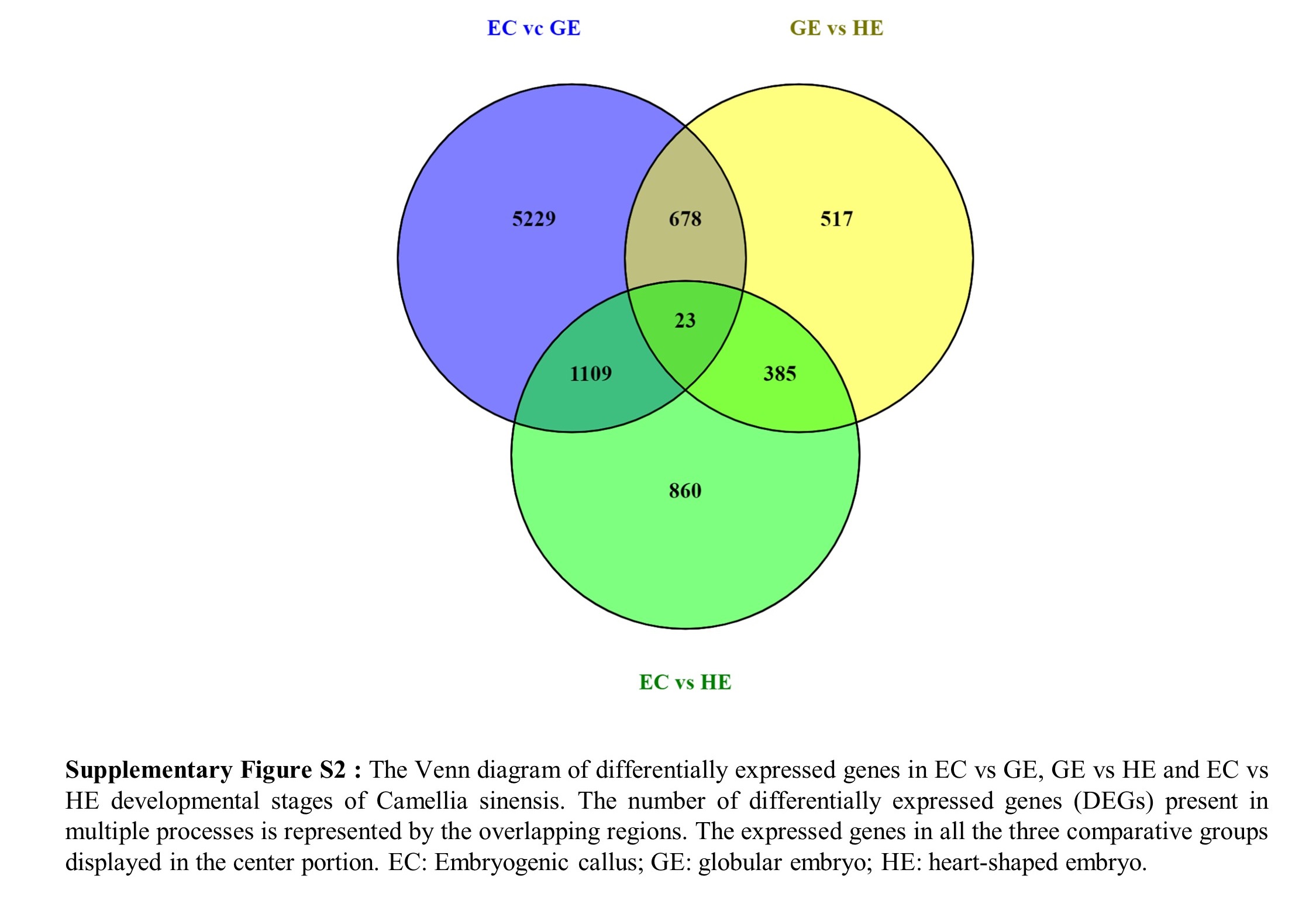

Supplement: Supplementary file 2 — Supplementary Material 2: Fig S2. The Venn diagram of differentially expressed genes in EC vs. GE, GE vs. HE and EC vs. HE developmental stages of Camellia sinensis. The number of Differentially expressed genes (DEGs) present in multiple processes is represented by the overlapping regions. The expressed genes in all the three comparative groups displayed in the center portion. EC: embryogenic callus; GE: globular embryo; HE: heart shaped embryo [file 12864_2024_10119_MOESM2_ESM.jpg]

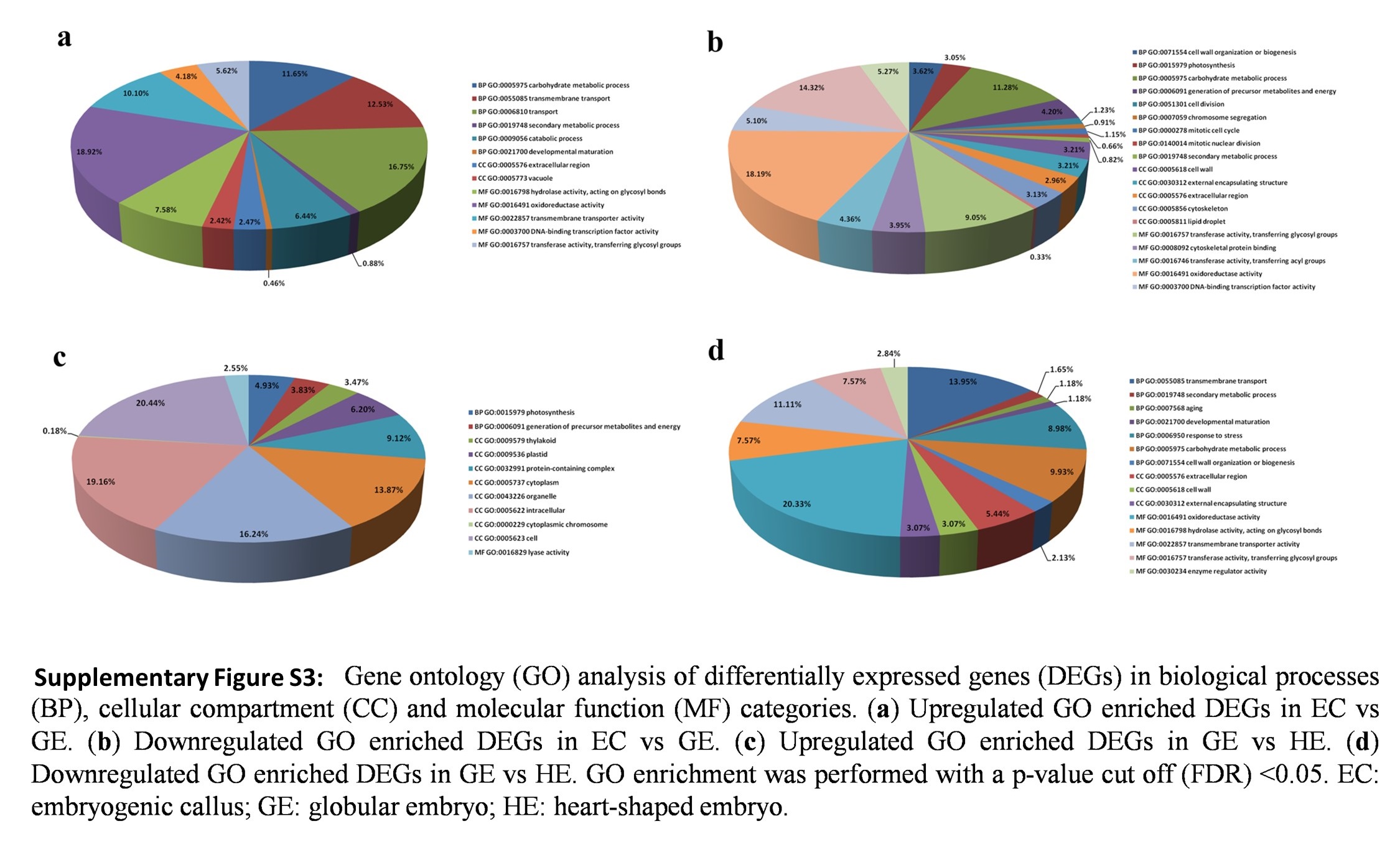

Supplement: Supplementary file 3 — Supplementary Material 3: Fig. S3. Gene ontology (GO) analysis of differentially expressed genes (DEGs) in biological processes (BP), cellular component (cc) and molecular function (MF) categories. (a) Upregulated GO enriched DEGs in EC vs GE. (b) Downregulated GO enriched DEGs in EC vs GE. (c) Upregulated GO enriched DEGs in GE vs. HE. (d) Downregulated GO enriched DEGs in GE vs. HE. Go enrichment was performed with a p-value cut off (FDR) <0.05. EC: embryogenic callus; GE: globular embryo; HE: heart shaped embryo [file 12864_2024_10119_MOESM3_ESM.jpg]
