# Supplementary material for: Integrated metabolomics and transcriptomics analysis highlight key pathways involved in the somatic embryogenesis of Darjeeling tea
Source: BMC Genomics. 2024 Feb 23;25:207. doi: 10.1186/s12864-024-10119-2 (PMC10893738; doi:10.1186/s12864-024-10119-2)
Supplement: Supplementary file 4 — Supplementary Material 4: Fig. S4. Multi-sequence alignment of amino acid sequences of similar genes [file 12864_2024_10119_MOESM4_ESM.pdf]

YUC1

Percent Identity Matrix - created by Clustal2.1

1:	Cluster-17835.140770	100.00	96.31
2:	Cluster-17835.140771	96.31	100.00

```
Cluster-17835.140770      MEMPEPAVIIVGAGPSGLATAACLNRLSIPNIVLEREDCFASLWKKKSYDCLHLHLAKQY  60
Cluster-17835.140771      MEMPEPAVIIVGAGPSGLATAACLNRLSIQNIVLEREDCFASLWKKKSYDCLHLHLAKQY  60
                          ***************************** ******************************

Cluster-17835.140770      CELPHMSYPKNSPTYVSKKQFIQYLDDYVAHFNISPMYNRPVVSADYDEVSQKWKVKVRN  120
Cluster-17835.140771      CELPHMSYPKNSPTYVSKKQFIQYLDDYVAHFNISPMYNRPVVSADYDEVSQKWKVKVRN  120
                          ************************************************************

Cluster-17835.140770      MVSDEVEEYSGKFMVVATGETSDEFIPEVEGLRNFTGEVIHSTLYKCGDKYRDKRVLVVG  180
Cluster-17835.140771      MVSDEVEEYSGKFMVVATGETSDEFIPEVEGLRNFTGEVIHSTLYKCGDKYRDKRVLVVG  180
                          ************************************************************

Cluster-17835.140770      SGNSGMEIAFDLSNYGAKTSIVVRSPLHIISREMAYLGLVLLKYIQVSIVDYLLVLLSKL  240
Cluster-17835.140771      SGNSGMEIAFDLSNYGAKTSIVVRSPLHIISREMAYLGLVLLKYIQVSIVDYLLVLLSKL  240
                          ************************************************************

Cluster-17835.140770      RYGDLTKYGIRRPEEGPFTLKVKYGKYPVIDVGTYSKIKSREIQVLPAITSIKGDEVVFQ  300
Cluster-17835.140771      RYGDLSKYGIQRPEEGPFTVKLKYGKYPVIDVGTCNKIKSREIQVLPAITSIKGDEVVFQ  300
                          *****:****:********:*:************ .************************

Cluster-17835.140770      NGKSHPFDAIVFATGFTRSTHKWLKGDNYLLNKDGLPKPSFPNHWKGQNGLYCAGLARRG  360
Cluster-17835.140771      NGKSHPFDAIVFATGFTRSTHKWLKGDNYLLNKDGLAKPSVPNHWKGQNGLYCCGLARRG  360
                          ************************************ ***.************.******

Cluster-17835.140770      LYGAAMDAQNIANDIKSAQ   379
Cluster-17835.140771      FYGAAMEAQNIANDVKSAL   379
                          :*****:*******:***
```

AAO1

```
Cluster-17835.65252    MPTILHPCSSILGILLLERESLAVPWLMMEERETAIRRRENLLFAVNGERFELSTVDPST   60
Cluster-17835.65253    ---------------------------MMEERETAIRRRENLLFAVNGERFELSTVDPST   33
                                                  *********************************

Cluster-17835.65252    TLLQFLRSQTRFKSVKLSCGEGGCGACVVLLSKYNPVDEEVENFTVSSCLTLLCSVDKCS   120
Cluster-17835.65253    TLLQFLRSQTRFKSVKLSCGEGGCGACVVLLSKYNPVDEEVENFTVSSCLTLLCSVDKCS   93
                       ************************************************************

Cluster-17835.65252    ITTTEGLGNSKDGFHPIHQRFAGFHASQCGFCTPGMCISLFSALINAEKAPRAEPPPGFS   180
Cluster-17835.65253    ITTTEGLGNSKDGFHPIHQRFAGFHASQCGFCTPGMCISLFSALINAEKAPRAEPPPGFS   153
                       ************************************************************

Cluster-17835.65252    KLTVSEAEKAIAGNLCRCTGYRPIVDACKSFAADVDMEDLGINSFWKKGESNEVKASKLP   240
Cluster-17835.65253    KLTVSEAEKAIAGNLCRCTGYRPIVDACKSFAADVDMEDLGINSFWKKGESNEVKASKLP   213
                       ************************************************************

Cluster-17835.65252    FYNPSDQICTFPEFLKNEIRSSMLLSSKSNSWYQPVNVKELMSMLLAENDTRVKLVVANT   300
Cluster-17835.65253    FYNPSDQICTFPEFLKNEIRSSMLLSSKSNSWYQPVNVKELMSMLLAENDTRVKLVVANT   273
                       ************************************************************

Cluster-17835.65252    GTGYYKEVDHYDKYIDLRHIPDLSMIRRDQMGIIIGATVTISKAILTLKEVNNGEFHSQG   360
Cluster-17835.65253    GTGYYKEVDHYDKYIDLRHIPDLSMIRRDQMGIIIGATVTISKAILTLKEVNNGEFHSQG   333
                       ************************************************************

Cluster-17835.65252    ELVFQKIANHMEKIASGFIRNSGSIGGNLVMAQRNYFPSDIATILLAVDSAVNIMTDLKH   420
Cluster-17835.65253    ELVFQKIANHMEKIASGFIRNSGSIGGNLVMAQRNYFPSDIATILLAVDSAVNIMTDLKH   393
                       ************************************************************

Cluster-17835.65252    EKLTLEEFLGRPPLDPRSVLLSIQIPCWETARNGSTKTDTKLLFETYRAAPRSLGNALAY   480
Cluster-17835.65253    EKLTLEEFLGRPPLDPRSVLLSIQIPCWETARNGSTKTDTKLLFETYRAAPRSLGNALAY   453
                       ************************************************************

Cluster-17835.65252    LNAAFLAEVTSCKTSNGVIIDNIQLAFGAYGTKHAIRARKVEECLAGKLLNIDVLFEAIK   540
Cluster-17835.65253    LNAAFLAEVTSCKTSNGVIIDNIQLAFGAYGTKHAIRARKVEECLAGKLLNIDVLFEAIK   513
                       ************************************************************

Cluster-17835.65252    LLEATIVPEIGTSRAAYRSSLAVGFLFEFLRPFIDVCGELTNGLVDGFNENQALSLSAKQ   600
Cluster-17835.65253    LLEATIVPEIGTSRAAYRSSLAVGFLFEFLRPFIDVCGELTNGLVDGFNENQALSLSAKQ   573
                       ************************************************************

Cluster-17835.65252    VVESNQKYYPVGEPITKSGAAIQASGEAVYVDDIPSPTNCLHGAFIYSTKPLARVKSVKI   660
Cluster-17835.65253    VVESNQKYYPVGEPITKSGAAIQASGEAVYVDDIPSPTNCLHGAFIYSTKPLARVKSVKI   633
                       ************************************************************

Cluster-17835.65252    KSESQQDGIAGVISYRDIPNGGSNIGAQAMFGPEPLFADDLTRCTGQALAFVVANTQKVA   720
Cluster-17835.65253    KSESQQDGIAGVISYRDIPNGGSNIGAQAMFGPEPLFADDLTRCTGQALAFVVANTQKVA   693
                       ************************************************************
```

Continued

AAO1

```
Cluster-17835.65252      DMAADTALVDYDTENLEPPILTLEEAVERSSFFVVPPFLNPAPVGDFSKGMAEADHKILS   780
Cluster-17835.65253      DMAADTALVDYDTENLEPPILTLEEAVERSSFFVVPPFLNPAPVGDFSKGMAEADHKILS   753
                         ************************************************************

Cluster-17835.65252      AKIKLGSQYYFYMETQTALAIPDEDNCMLVYSSSQCPEFAHASIASCLGIPEHNVRVITR   840
Cluster-17835.65253      AKIKLGSQYYFYMETQTALAIPDEDNCMLVYSSSQCPEFAHASIASCLGIPEHNVRVITR   813
                         ************************************************************

Cluster-17835.65252      RVGGGFGGKAIRAMPVATACALAAHKLQCPVRIYLNRKTDMVMSGGRHPMKITYSVGFKS   900
Cluster-17835.65253      RVGGGFGGKAIRAMPVATACALAAHKLQCPVRIYLNRKTDMVMSGGRHPMKITYSVGFKS   873
                         ************************************************************

Cluster-17835.65252      SGKITALHLDILINGGLSVDISPILPLNMLGALKKYDWGALSFDFKVCKTNHSTKSAMRA   960
Cluster-17835.65253      SGKITALHLDILINGGLSVDISPILPLNMLGALKKYDWGALSFDFKVCKTNHSTKSAMRA   933
                         ************************************************************

Cluster-17835.65252      PGEVQASFIAESVIEHVASVLSMEVDSVRNKNLHTFDSLNIFYEGSAGESVEYTLPSIWD   1020
Cluster-17835.65253      PGEVQASFIAESVIEHVASVLSMEVDSVRNKNLHTFDSLNIFYEGSAGESVEYTLPSIWD   993
                         ************************************************************

Cluster-17835.65252      KLARSSCLHQRIEMIQHFNMCNKWRKRGISRVPIIHEVIVRPTPGKVSILRDGSIVVEVG   1080
Cluster-17835.65253      KLARSSCLHQRIEMIQHFNMCNKWRKRGISRVPIIHEVIVRPTPGKVSILRDGSIVVEVG   1053
                         ************************************************************

Cluster-17835.65252      GIELGQGLWTKVKQMTAFALSMIQCDGTEELLKKVRVIQSDTLSLIQGGFTAGSTTSESS   1140
Cluster-17835.65253      GIELGQGLWTKVKQMTAFALSMIQCDGTEELLKKVRVIQSDTLSLIQGGFTAGSTTSESS   1113
                         ************************************************************

Cluster-17835.65252      CEAVRLCCNILVERLIPLKEKLEEQTGSIKWEALILQAHLQAVNLSTSSFYVPDFTSMQY   1200
Cluster-17835.65253      CEAVRLCCNILVERLIPLKEKLEEQTGSIKWEALILQAHLQAVNLSTSSFYVPDFTSMQY   1173
                         ************************************************************

Cluster-17835.65252      LNYGVAVSEVEVNLLTGETSILQADIIYDCGQSMNPAVDLGQVEGAFVQGVGFFMFEEYL   1260
Cluster-17835.65253      LNYGVAVSEVEVNLLTGETSILQADIIYDCGQSMNPAVDLGQVEGAFVQGVGFFMFEEYL   1233
                         ************************************************************

Cluster-17835.65252      TNSDGLVVSDGTWTYKIPTVDNIPKQFNVELLNSGHHQNRVLSSKASGEPPLLLAVSVHC   1320
Cluster-17835.65253      TNSDGLVVSDGTWTYKIPTVDNIPKQFNVELLNSGHHQNRVLSSKASGEPPLLLAVSVHC   1293
                         ************************************************************

Cluster-17835.65252      ATRAAIKEARKQLSSWKGLDGSNSTFQLEVPATMPVVKEFCGLDSVEWYLKSLH--       1374
Cluster-17835.65253      ATRAAIKEARKQLSSWKGLDGSNSTFQLEVPATMPVVKEFCGLDIVEWYLKSLLSS       1349
                         ******************************************** ********
```

Percent Identity Matrix - created by Clustal2.1

```
1: Cluster-17835.65252   100.00   99.85
2: Cluster-17835.65253    99.85  100.00
```

IAA26

```
Percent Identity  Matrix - created by Clustal2.1

     1: Cluster-17835.41237   100.00   59.85    -nan
     2: Cluster-17835.89098    59.85  100.00   85.00
     3: Cluster-17835.41233     -nan   85.00  100.00
```

```
Cluster-17835.41237      ----MENKDEDCPQLLDLIPKNREWIVQRGDRHRSHGSSEEKKLELRLGPPGEREEQDWT    56
Cluster-17835.89098      MEGCSNKDEVACPQLLDLIPKDREWLLQRDE-QGSHGYSEEKKLELRLGPPGEG---DWT    56
Cluster-17835.41233      ------------------------------------------------------------    0

Cluster-17835.41237      KN-------NNSRERGGSLSSVGYFSNMASLTHNNGGNRVENGWIN------NQKVSFAD    103
Cluster-17835.89098      IKNGTDQNNSTEREREDSILSLGYFSNMKTV---------ENSWIMNNNGNQNHKFSFSE    107
Cluster-17835.41233      ------------------------------------------------------------    0

Cluster-17835.41237      NPVGKTL--KTPWSSSPFIHQIQTNNP-----------------------QSSQTCGTKG    138
Cluster-17835.89098      NPIGITALSSPSWSSSTFH-AIQTQQQQRQQQQQFLQLQSNPHGLPVMAKESSQPCHS-K    165
Cluster-17835.41233      ------------------------------------------------------------    0

Cluster-17835.41237      GVDLQKSAEKKAFS----------------------------------------------    152
Cluster-17835.89098      PVDLQNAAEKKAFSPASANTAVPNSSQKRTAPASVVGWPPIRSFRKNLASSCSSKPTSES    225
Cluster-17835.41233      ------------------------------------------------------------    0

Cluster-17835.41237      ------------------------------------------------------------    152
Cluster-17835.89098      QNLVPIEVPNHKLVESCRKDFFVKINMDGVPIGRKVDLKAYDSYEKLSSAVDDLFRGLLA    285
Cluster-17835.41233      --------------------------MDGVPIGRKVDLKAYDSYEKLSSSVDELFRGLLA    34

Cluster-17835.41237      ------------------------------------------------------------    152
Cluster-17835.89098      AQRDSSPGGIENKQEAEKAITGLLDGSGEYTLVYEDNEGDRILVGDVPWHMFVSTVKRLR    345
Cluster-17835.41233      AQRNSSAGGIQNKHEGERAITDLLDGSGEYTLVYEDNEGDRMLVGDVPWHMFLSTVKRLR    94

Cluster-17835.41237      --------------------------      152
Cluster-17835.89098      VLKSSELSTLCLGSKKETIQVDVGSK      371
Cluster-17835.41233      VLKSSELSTLRFGSKQEKIQLDAASK      120
```

SAUR32

Percent Identity Matrix - created by Clustal2.1

1: Cluster-17835.128969	100.00	9.80	23.81
2: Cluster-17835.2512	9.80	100.00	32.43
3: Cluster-17835.19385	23.81	32.43	100.00

```
Cluster-17835.128969      --------MMSIYSSLWLQF-----------------TQVLE-------ANAAIKKLKKQ  28
Cluster-17835.2512        --------------------------MGSGDKSLLSFHQHQH------HHHHRHQGKKQE  28
Cluster-17835.19385       MDVINPKAKKGLITKTWERCRSIGGGGGGGSGALMMKSRSLPRPGSGGGGGAAVEDEEKQ  60
                                                                :               :  :::

Cluster-17835.128969      PQ--------------------------------NPIAQAILELGGSTLAYLSS------  50
Cluster-17835.2512        EEVVRDVPKGCLAIKVGQEEEEQQRFVVPVMYFNHPRFMQLLKEAEEEYGFDQKGTITIP  88
Cluster-17835.19385       PRKHRVAPEGCFSVYVGPQ---KQRFVVKTEYANHPLFKMLLEEAELEYGFNSDGPLALP  117
                           .                                :*    :*: .    .: ..

Cluster-17835.128969      NNLQLFFKFLWQN-------------------------------  63
Cluster-17835.2512        CHVEEFRYVQGMIDEELHHHHHLCWLF-----------------  115
Cluster-17835.19385       CNVDRFYKVLVEMEDSYEN-RGGCTGFGSYRLLSPYRLVAMNKL  160
                           ::: *  .
```

SAUR50

```
Percent Identity  Matrix - created by Clustal2.1

1: Cluster-17835.108846   100.00  100.00   23.73
2: Cluster-17835.108853   100.00  100.00   23.73
3: Cluster-10437.1         23.73   23.73  100.00
```

```
Cluster-17835.108846      MKVAKLT-KLKSALKKWPSFTKLD------------------RT-----ISS-STSVTA  34
Cluster-17835.108853      MKVAKLT-KLKSALKKWPSFTKLD------------------RT-----ISS-STSVTA  34
Cluster-10437.1           MESAKVQTKKNLFVKTWERCRSIPGGREKKSPSKPSSIFSKSKSCHYTTTKSSSKDDNQR  60
                          *: **:  * :  :*.*    .:                         ** . .

Cluster-17835.108846      TDDGAATAAGNLHPVYVGKSRRLYLVSTEVIDHPLFQVLVDKSNGSD------GITVACE  88
Cluster-17835.108853      TDDGAATAAGNLHPVYVGKSRRLYLVSTEVIDHPLFQVLVDKSNGSD------GITVACE  88
Cluster-10437.1           NTKSRQVAPEGCFSVYVGPEKQRFVIKTKFANHPLFRMLLEDAEMEYGYNSEGPLLLPCE  120
                          . ..  .*   . . ****  .:: :::.*:. :****::*::.:: .        : : **

Cluster-17835.108846      VVLFDHLLWMLENAGADV------ASV-----------DELVEFYS-Y----------  118
Cluster-17835.108853      VVLFDHLLWMLENAGADV------ASV-----------DELVEFYS-Y----------  118
Cluster-10437.1           VDLFYKVLAEMDSKEIVPSCGFAYGSCSPFNPSRRLGNNNNMRKVYGSYGLLTPSRLLKM  180
                          * ** ::*  ::.            .*              ::: :.*. *

Cluster-17835.108846      ---   118
Cluster-17835.108853      ---   118
Cluster-10437.1           NHF   183
```

ABCB4

```
Percent Identity  Matrix - created by Clustal2.1

     1: Cluster-17835.13084  100.00   87.29
     2: Cluster-17835.13079   87.29  100.00
```

```
Cluster-17835.13084      MGFLSLLEVASMPILQVLIISILGAFMATDYLNLLPTASRKSLNKIVFMVFTPSLMFASL    60
Cluster-17835.13079      MGFLSLLEVASMPILQVLIISILGAFMATDYLNLLPTASRKSLNKIVFMVFTPSLMFASL    60
                         ************************************************************

Cluster-17835.13084      AETVTFQDLIAWWFMPINVGLTFLFGGILGWIAVKILKPKPHLEGLIIATCSSGNLGNLM    120
Cluster-17835.13079      AETVTFQDLIAWWFMPINVGLTFLFGGILGWIAVKILKPKPHLEGLIIATCSSGNLGNLM    120
                         ************************************************************

Cluster-17835.13084      LIVVPAICSEEGSPFGNHKACSSVALSYASFSMALGGFFIWTYTYQLIRSSSMKYKALQA    180
Cluster-17835.13079      LIVVPAICSEEGSPFGNHKACSSVALSYASFSMAVRQFCMLYTHNSML-----IFQ---I    172
                         **********************************:  * :     .::      ::

Cluster-17835.13084      ADEISKLPNNDLESDEKTHLLEGEVQEHVAILVPSTRPTEEYTEENQAMPQVSASKLEKE    240
Cluster-17835.13079      QNHASKTDN---------------------------------------------------    181
                          :. **  *

Cluster-17835.13084      NVSFWGNIIVVLHQLLEELLAPPTLSAIVGFFFGAIPWLKNLIIGESAPLRVIQDSIKLL    300
Cluster-17835.13079      ------------------------------------------------------------    181

Cluster-17835.13084      GGGTIPCITLILGGNLTQGLRTARLKPIMIIAVICVRYVLLPFIGIGVVRAASSLGFLPS    360
Cluster-17835.13079      ------------------------------------------------------------    181

Cluster-17835.13084      DPLYHYVLMIQFALPPAMNIGTMTQLFDVAQEECSVLFLWTYLVACLSLTVWSTIFMWTL    420
Cluster-17835.13079      ------------------------------------------------------------    181

Cluster-17835.13084      S       421
Cluster-17835.13079      -       181
```

A-ARR

Percent Identity Matrix - created by Clustal2.1

1:	Cluster-17835.76446	100.00	21.58
2:	Cluster-17835.29790	21.58	100.00

```
Cluster-17835.76446      --MITT--GISLKVDDSFLS----------NSYPVIPAIESDGALLSIQTEILQSSASTL    46
Cluster-17835.29790      MGMATESQFHVLAVDDSLIDRKLIERLLKTSSYQVTT-VD-------------SGNK--    43
                           * *      * ****::.          .** *   ::              *. .

Cluster-17835.76446      LFSILYIGIFSVAHSAHSHSHAAAPAPAVDCTTLVLKMTDCLSYVTNGSTVKKPEGT-CC    105
Cluster-17835.29790      --ALKFLGLQEDDQTNPNHQPSFSPNNNQEVEVNLIITDYCMPGMTGYDLLKKIKESSSL    101
                           :: ::*: .  ::  .*. : :*    :   . ::    *:  :*. . :** : : .

Cluster-17835.76446      SVLKTVLTTNEAL---RDTSLAGGAA----------------------------------    128
Cluster-17835.29790      KNIPVVIMSSENVPARINRCLEEGAEEFFLKPVRLADVSKLKTHMLKTKFKNHQKQESQE    161
                         . : .*: :.* :    : .*  **

Cluster-17835.76446      ----AEELRV-------------AAFLLQDGVSSDYLHDESVTIGSRFQLINPHILPWV    170
Cluster-17835.29790      SSETEEEDQQEDIQQQQQQSSNNNKRKAMEEGLSTDRTRP-------RY---------S    204
                              ** :                   :::*:*:*  :        *:

Cluster-17835.76446      GTISKPTILLPWVG 184
Cluster-17835.29790      GLT----VV----- 209
                         *      ::
```

Supplementary figure S4. Multi-sequence alignment of amino acid sequences of similar genes.
