# Supplementary material for: Integrated metabolomics and transcriptomics analysis highlight key pathways involved in the somatic embryogenesis of Darjeeling tea
Source: BMC Genomics. 2024 Feb 23;25:207. doi: 10.1186/s12864-024-10119-2 (PMC10893738; doi:10.1186/s12864-024-10119-2)
Supplement: Supplementary file 9 — Supplementary Material 9: Tables S5-S6. Summary for the clean reads mapped to the Trinity-assembled transcriptome and summary statistics of transcriptome annotation [file 12864_2024_10119_MOESM9_ESM.docx]

**Table S5** Summary for the clean reads mapped to the Trinity-assembled transcriptome

| Sample name | Clean reads | Total mapped |
| --- | --- | --- |
| EC1 | 23,263,342 | 17,790,731 (76.48%) |
| EC2 | 24,143,045 | 20,050,401 (83.05%) |
| GE1 | 23,934,097 | 20,048,938 (83.77%) |
| GE2 | 20,833,214 | 17,328,767 (83.18%) |
| HE1 | 23,035,743 | 19,895,301 (86.37%) |
| HE2 | 24,715,949 | 21,463,198 (86.84%) |
| Average | 23,320,898 | 19,429,556(83.28%) |

**Table S6** Summary statistics of transcriptome annotation

|  | Number of Unigenes | Percentage (%) |
| --- | --- | --- |
| Annotated in NR | 118,158 | 67.66 |
| Annotated in NT | 102,520 | 58.70 |
| Annotated in KO | 44,556 | 25.51 |
| Annotated in Swissport | 90,628 | 51.90 |
| Annotated in PFAM | 77,422 | 44.33 |
| Annotated in GO | 65,489 | 37.50 |
| Annotated in KOG | 28,546 | 16.35 |
| Annotated in all Databases | 15,615 | 8.94 |
| Annotated in at least one Database | 130,006 | 74.44 |
| Total Unigenes | 174,637 | 100 |
